# Temporal dynamics and determinants of early recurrence after curative resection for stage I-III rectal cancer: integrated analyses of hazard function, survival, and competing risks

**DOI:** 10.3389/fonc.2026.1850553

**Published:** 2026-06-18

**Authors:** Rongda He, Zhiqiang Zhang, Ruishu Li, Yulong He

**Affiliations:** 1 Guangdong Provincial Key Laboratory of Digestive Cancer Research, The Seventh Affiliated Hospital, Sun Yat-sen University, Shenzhen, China; 2 Gastrointestinal Surgery, Digestive Diseases Center, The Seventh Affiliated Hospital, Sun Yat-sen University, Shenzhen, China

**Keywords:** competing risks, early recurrence, lymph node ratio, rectal cancer, temporal dynamics, time-to-recurrence

## Abstract

**Background:**

Recurrence after curative resection remains a major determinant of long-term outcome in rectal cancer. However, the temporal pattern of postoperative recurrence, the determinants of early recurrence, and their relationships with survival and recurrence patterns have not been fully characterized. This study aimed to define the time distribution of recurrence after curative surgery for stage I-III rectal cancer, identify factors associated with early recurrence, and examine their links with long-term outcomes and recurrence patterns.

**Methods:**

We retrospectively included 1,045 patients with stage I-III rectal cancer who underwent curative surgery between January 1, 2014, and April 30, 2019. Smoothed hazard curves were used to identify the high-risk window for early recurrence. Logistic regression in an adequate-follow-up cohort was used to analyze early recurrence, Cox regression to evaluate time-to-recurrence (TTR) and overall survival (OS), and cumulative incidence functions with Fine-Gray models to assess local recurrence and distant metastasis.

**Results:**

Recurrence hazard peaked at approximately 24 months after surgery. With recurrence within 24 months defined as early recurrence, multivariable logistic regression identified lymph node ratio (LNR; per 0.1 increase: OR = 1.36, 95% CI 1.18-1.57, P < 0.001) and T4 stage (OR = 2.74, 95% CI 1.13-6.65, P = 0.026) as independent correlates. In multivariable Cox analyses, LNR, T4 stage, and carcinoembryonic antigen were independently associated with worse TTR, whereas LNR, T4 stage, carcinoembryonic antigen, and poor/undifferentiated differentiation were independently associated with worse OS. Distant metastasis consistently exceeded local recurrence during follow-up. Fine-Gray analysis showed that LNR (per 0.1 increase: sHR = 1.25, 95% CI 1.13-1.38, P < 0.001) and T4 stage (sHR = 4.22, 95% CI 1.77-10.05, P = 0.001) were independently associated with distant metastasis; the local-recurrence model was exploratory because of limited events.

**Conclusions:**

Recurrence after curative resection for rectal cancer showed clear temporal dynamics and peaked at approximately 24 months. LNR and T4 stage were the most consistent correlates across early recurrence, long-term outcomes, and distant metastasis, supporting further evaluation of risk-stratified postoperative surveillance based on time window and pathological risk burden.

## 1 Introduction

Recurrence after curative resection remains one of the key clinical determinants of long-term prognosis in rectal cancer (1, 2). Although total mesorectal excision, perioperative treatment, and multidisciplinary management have continued to improve, the risk of postoperative failure has not been completely eliminated, and once local recurrence or distant metastasis occurs, subsequent treatment pathways and survival outcomes are often substantially altered (1–3). In postoperative management, however, the clinically relevant question is not merely whether recurrence will occur, but whether recurrence tends to cluster within a particular time window and which patients are more likely to fail during that period. The answers to these questions directly determine whether postoperative surveillance should follow a one-size-fits-all approach or be stratified according to time windows and pathological risk burden (1, 2).

Previous studies on postoperative outcomes in rectal cancer have largely treated recurrence as a static endpoint and have primarily focused on identifying correlates of recurrence or death as binary events (2, 4). Although this approach is useful for overall risk stratification, it usually provides limited insight into the temporal distribution of recurrence risk during follow-up (4, 5). If recurrence is concentrated within a specific postoperative phase, then when recurrence occurs may be as important as whether recurrence occurs, and surveillance strategies based on dynamic risk assessment may be more clinically relevant (1, 5, 6).

Accordingly, early recurrence has become a clinically meaningful concept in postoperative management. However, in rectal cancer, the definition of early recurrence remains inconsistent, with substantial variation in the time cutoffs used across studies, and many cutoffs are based primarily on convention rather than on the actual time distribution of recurrence and subsequent outcome differences (7–9). This definitional heterogeneity not only hampers comparability across studies but also limits the clinical interpretability of early recurrence (7, 8). Therefore, if the dynamic pattern of postoperative recurrence risk can first be characterized over time and the high-risk recurrence window then defined accordingly, the resulting definition of early recurrence may better reflect the real clinical course (7, 8).

Beyond recurrence timing, postoperative failure risk should not be summarized by a single endpoint alone. In patients with rectal cancer, at least three related but non-identical questions exist: whether recurrence clusters over time, which factors predispose patients to recurrence within the high-risk window, and whether these same factors also affect long-term outcomes and recurrence patterns (4, 10, 11). Existing studies usually focus separately on early recurrence, overall survival, or local versus distant failure, and relatively few have integrated these dimensions within a unified analytical framework (11, 12). Thus, whether factors such as T stage, nodal involvement, tumor differentiation, and tumor markers extend across the continuum of recurrence timing, long-term outcomes, and failure patterns remains insufficiently characterized (4, 10, 11).

Accordingly, in a cohort of patients with stage I-III rectal cancer who underwent curative resection, this study first used smoothed hazard curves to depict the temporal dynamics of postoperative recurrence risk and thereby define the high-risk window for early recurrence. We then combined logistic regression, Cox regression, and competing-risk analysis to evaluate correlates of early recurrence, long-term survival outcomes, and local recurrence versus distant metastasis, respectively. Through this integrated analytical framework, the present study aimed to address three interrelated clinical questions within a single research logic: whether recurrence exhibits temporal clustering, which patients are more likely to recur within the high-risk window, and through which failure pattern risk is primarily manifested in high-risk patients. In doing so, we sought to provide evidence for postoperative risk-stratified surveillance based on time windows and pathological risk burden.

## 2 Methods

### 2.1 Study population

This was a single-center retrospective cohort study that included patients with rectal cancer who underwent surgery at Sun Yat-sen University Affiliated Hospital between January 1, 2014, and April 30, 2019. The original database contained 1,182 patients, of whom 1,045 were ultimately included after screening.

Inclusion criteria were as follows: (1) postoperative pathology confirming rectal adenocarcinoma, mucinous adenocarcinoma, or signet-ring cell carcinoma; (2) tumor lower margin located 0–20 cm above the dentate line; (3) receipt of curative surgery; (4) stage I-III disease according to the 8th edition of the AJCC staging system; (5) first presentation and completion of surgical treatment at our center; (6) complete clinicopathological and follow-up data; and (7) postoperative survival and follow-up duration ≥ 3 months.

Exclusion criteria were as follows: (1) stage IV disease; (2) non-curative surgery; (3) missing clinicopathological data; (4) follow-up < 3 months; and (5) non-initial presentation or previous surgical treatment.

**Figure 1:**
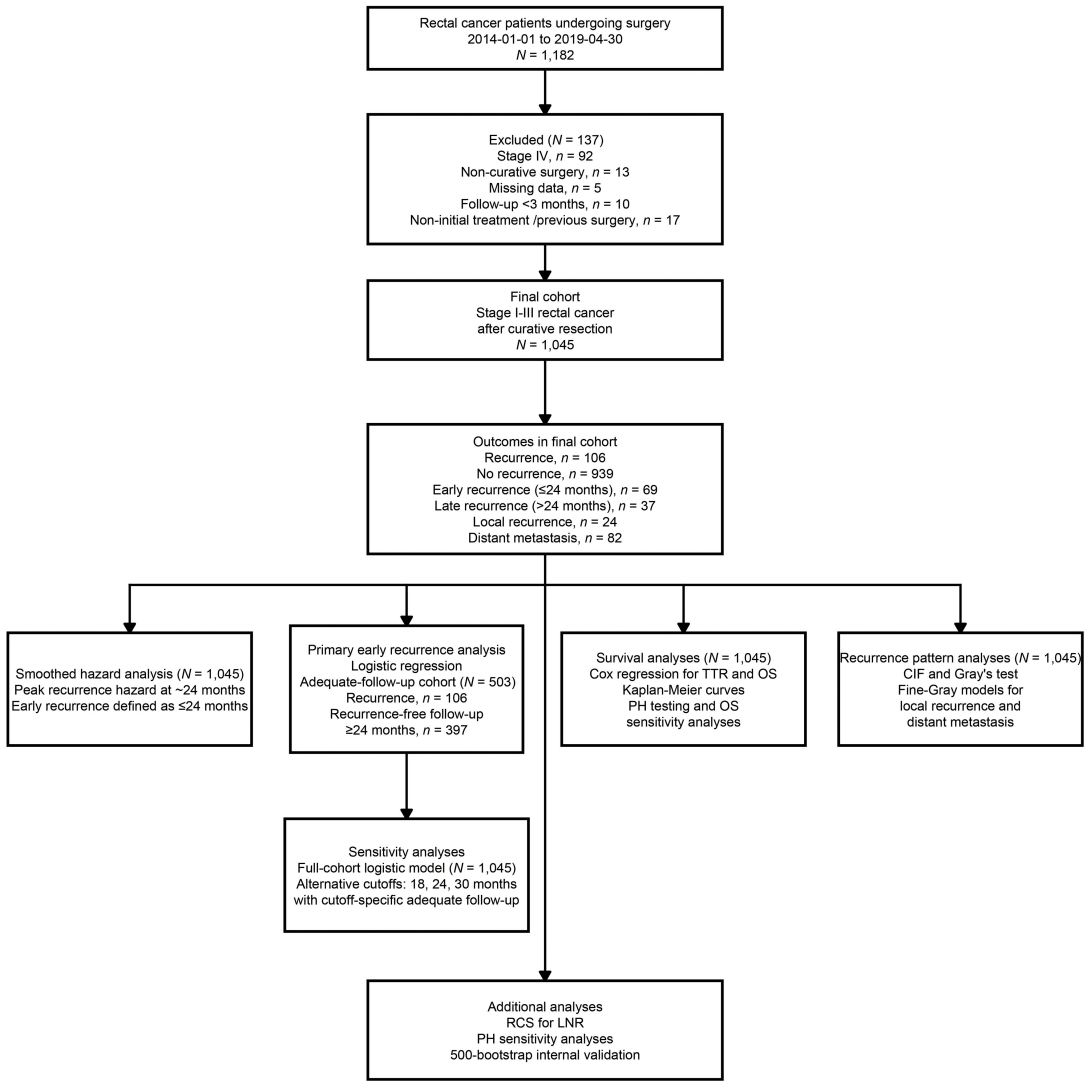
Patient selection and analytic workflow of the study cohort. Among 1,182 patients with rectal cancer undergoing surgery between January 1, 2014, and April 30, 2019, 1,045 patients with stage I-III disease who underwent curative resection were ultimately included after predefined exclusions. The flowchart summarizes patient selection, recurrence outcomes, and the overall analytic framework. The primary early-recurrence logistic analysis was performed in the adequate-follow-up cohort, which included all patients with documented recurrence and recurrence-free patients with follow-up of at least 24 months. Full-cohort and alternative-cutoff analyses were retained as sensitivity analyses. Survival outcomes and recurrence patterns were analyzed in the full cohort using Cox regression, Kaplan-Meier curves, cumulative incidence functions, and Fine-Gray models.

A total of 137 patients were excluded, including 92 with stage IV disease, 13 who underwent non-curative surgery, 5 with missing clinicopathological data, 10 with follow-up < 3 months, and 17 who were not initial presentations or had undergone previous surgery. The overall study workflow, including patient screening, exclusions, and the analytical framework, is shown in **Figure 1**.

This study was reported in accordance with the Strengthening the Reporting of Observational Studies in Epidemiology (STROBE) statement.

### 2.2 Clinicopathological variables

Demographic characteristics, clinicopathological features, treatment information, and follow-up outcomes were collected. Continuous variables included age, body mass index (BMI), maximum tumor diameter, distance from the dentate line, and lymph node ratio (LNR). Categorical variables included sex, histological type, tumor differentiation, lymphovascular invasion (LVI), surgical approach, sphincter preservation, performance of total mesorectal excision (TME), carcinoembryonic antigen (CEA), carbohydrate antigen 19-9 (CA19-9), neoadjuvant therapy, adjuvant chemotherapy, T stage, N stage, and AJCC stage.

Histological type was categorized as adenocarcinoma versus mucinous adenocarcinoma/signet-ring cell carcinoma, and tumor differentiation was categorized as well/moderately differentiated versus poor/undifferentiated (13). CEA was stratified at 5 ng/mL and CA19–9 at 35 U/mL. T stage was grouped as T1-2, T3, and T4, and N stage as N0, N1, and N2. LNR was defined as the number of positive lymph nodes divided by the total number of examined lymph nodes and was entered into the regression models as a continuous variable on its original 0–1 scale; previous studies have suggested that LNR is an important indicator of regional nodal tumor burden (10). For plotting survival curves and cumulative incidence curves, LNR was further categorized into three groups: LNR = 0, 0 < LNR ≤ 0.143, and LNR > 0.143, where 0.143 represented the median LNR among patients with LNR > 0. This grouping was used only for graphical stratification and was not intended as a prespecified clinical threshold. All patients had at least one examined lymph node. For clinical interpretability, regression estimates for LNR are presented per 0.1 absolute increase in the main and supplementary tables, whereas LNR was retained on its original scale for graphical stratification and spline visualization.

### 2.3 Follow-up and endpoints

Follow-up was censored on December 31, 2019. The primary endpoints included time-to-recurrence (TTR), overall survival (OS), early recurrence, and recurrence patterns. TTR was defined as the interval from surgery to the first imaging- or pathology-confirmed tumor recurrence. Patients without documented recurrence were censored at death or last follow-up, whichever occurred first. TTR Event was coded as 1 only when true local recurrence or distant metastasis occurred; death without documented recurrence was treated as censoring rather than as a recurrence event. Accordingly, this endpoint should be interpreted as recurrence-focused TTR rather than disease-free survival. OS was defined as the interval from the date of surgery to death from any cause or the last follow-up (2).

Early recurrence was defined as documented recurrence occurring within 24 months after curative resection. For the primary logistic regression analysis of early recurrence, we used an adequate-follow-up cohort to reduce potential outcome misclassification. This cohort included all patients with documented recurrence and recurrence-free patients with follow-up of at least 24 months. Patients with recurrence after 24 months and recurrence-free patients with follow-up of at least 24 months were classified as non-early recurrence, whereas recurrence-free patients with follow-up shorter than 24 months were excluded from the primary logistic analysis. A full-cohort logistic analysis was retained as a sensitivity analysis. Recurrence patterns were categorized as local recurrence and distant metastasis. Local recurrence was strictly defined as anastomotic recurrence or pelvic local recurrence, whereas distant metastasis included all distant metastatic sites. If a patient developed local recurrence first and later developed distant metastasis, the patient was classified as local recurrence and TTR Months was recorded at the time of local recurrence. If local recurrence and distant metastasis occurred simultaneously, the patient was classified as distant metastasis. In the competing-risk analysis, local recurrence and distant metastasis were treated as mutually competing types of failure.

### 2.4 Statistical analysis

All statistical analyses were performed using R software (version 4.5.2; R Foundation for Statistical Computing, Vienna, Austria).

Continuous variables are presented as median (interquartile range [IQR]) and were compared using the Wilcoxon rank-sum test. Categorical variables are presented as frequencies and column percentages and were compared using Pearson’s chi-square test; for 2×2 tables, Yates’ continuity correction was applied, and Fisher’s exact test was used when expected cell counts were small. Baseline characteristics were compared between the recurrence and non-recurrence groups.

The temporal dynamics of postoperative recurrence risk were described using a smoothed hazard curve, on the basis of which 24 months after surgery was defined as the primary data-driven cutoff for early recurrence in this cohort. The 24-month cutoff was considered exploratory and data-informed rather than a universal threshold. Alternative cutoff analyses using 18, 24, and 30 months were performed to assess the robustness of the early-recurrence findings. Hazard smoothing was performed using a nonparametric B-spline approach to estimate changes in recurrence risk over time in a data-driven manner (14). The smoothing procedure used the full cohort with TTR months as the time scale and TTR event as the recurrence indicator, with patients without recurrence treated as right-censored at death or last follow-up.

Univariable and multivariable logistic regression analyses were performed to identify correlates of early recurrence in the adequate-follow-up cohort. The final multivariable model included five variables: LNR, T stage, CEA, tumor differentiation, and adjuvant chemotherapy. Variables were selected on the basis of a univariable *P* value < 0.10, together with clinical judgment and the principle of minimizing collinearity. Because AJCC stage is collinear with T and N stage, it was not included in the logistic model. Given that LNR reflects nodal tumor burden as a continuous measure and is closely related to N stage, LNR was retained in the multivariable model without simultaneous inclusion of N stage to reduce collinearity while preserving more granular prognostic information. Adjuvant chemotherapy was included as an adjustment variable to account for treatment allocation differences associated with baseline risk; however, given the retrospective design and the potential for treatment indication bias, its estimated association was interpreted only as an adjusted association rather than a causal treatment effect. Pairwise Spearman rank correlations among candidate predictors were additionally calculated to descriptively assess potential collinearity, particularly among AJCC stage, T stage, N stage, and LNR. Endpoint-specific covariate sets were constructed according to univariable screening, clinical relevance, collinearity assessment, and event-number considerations, with the detailed rationale provided in the **Supplementary Materials**. Although LNR was entered into regression models on its original 0–1 scale, regression estimates for LNR were rescaled to represent each 0.1 absolute increase for clinical interpretability using the transformation exp[0.1 × log(estimate)]. The same transformation was applied to confidence interval limits, and *P* values were unchanged. For logistic regression models, confidence intervals and *P* values were both derived using Wald-based inference to ensure consistency between interval estimates and significance testing. To evaluate whether the primary findings were robust to additional adjustment for LVI, an extended multivariable logistic model including LVI was fitted in the adequate-follow-up cohort as a supplementary analysis.

Univariable and multivariable Cox proportional hazards regression analyses were used to evaluate correlates of TTR and OS. The multivariable models for both TTR and OS included seven variables: maximum tumor diameter, tumor differentiation, T stage, LVI, LNR, CEA, and adjuvant chemotherapy. Variable selection followed the same principles described above. AJCC stage was not included in the Cox models. The proportional hazards assumption was evaluated using Schoenfeld residuals through the cox.zph function (15). Additional sensitivity analyses, including a stratified Cox model and a time-varying Cox model, were conducted to address mild non-proportional hazards in the OS model. To assess the potential influence of dependent censoring in the recurrence-focused TTR analysis, an additional competing-risk sensitivity analysis was performed in which death without documented recurrence was treated as a competing event for recurrence.

Kaplan-Meier methods were used to plot TTR and OS curves, and between-group differences were compared using the log-rank test. The cumulative incidences of local recurrence and distant metastasis were estimated using cumulative incidence functions (CIFs) and compared using Gray’s test. Fine-Gray subdistribution hazard models were further used to evaluate correlates of the competing risks of local recurrence and distant metastasis. Because only a limited number of local recurrence events occurred, the multivariable local-recurrence model was interpreted as exploratory.

Restricted cubic spline (RCS) models were used to evaluate the associations of LNR with early recurrence, TTR, and OS, with the aim of exploring potential nonlinearity rather than deriving a clinical threshold (16). The primary early-recurrence spline model was fitted in the adequate-follow-up cohort, and the full-cohort spline model was retained as a sensitivity analysis. RCS knots for LNR were selected using a prespecified robust algorithm designed to handle the zero-inflated distribution of LNR, and the same knot locations were used across endpoints to ensure comparability. Internal validation was also performed. For the primary early-recurrence logistic model in the adequate-follow-up cohort, discrimination and calibration were assessed; for the Cox models of TTR and OS, the C-index and calibration were evaluated. Internal validation was conducted using 500 bootstrap resamples (17). Exploratory decision-curve analyses were additionally performed for the primary adequate-follow-up early-recurrence logistic model and the full-cohort sensitivity model to assess potential clinical utility across a range of threshold probabilities.

Several sensitivity analyses were additionally performed. First, the original full-cohort logistic model was retained as a sensitivity analysis in which all patients without documented recurrence were classified as non-early recurrence regardless of follow-up duration. Second, alternative-cutoff analyses were performed using 18, 24, and 30 months under the adequate-follow-up framework, with recurrence-free patients censored before the corresponding cutoff excluded. Third, full-cohort alternative-cutoff analyses were retained as supplementary sensitivity analyses. Additional descriptive analyses were performed to characterize follow-up distribution, LVI distribution according to year of surgery, and observed recurrence rates stratified by T stage and AJCC stage.

To assess potential selection associated with the adequate-follow-up restriction, baseline characteristics were compared between the adequate-follow-up cohort and recurrence-free patients excluded because of follow-up shorter than 24 months. A recurrence-free-only comparison between retained recurrence-free patients and excluded recurrence-free patients was also performed.

All tests were two-sided, and P < 0.05 was considered statistically significant. No missing data were present for any included variable.

### 2.5 Ethics statement

This study was approved by the Medical Ethics Committee of the Seventh Affiliated Hospital of Sun Yat-sen University, Shenzhen (Acceptance No. KY-2025-351; Approval No. KY-2025-351-01). Because this was a retrospective study using de-identified data, the requirement for informed consent was waived in accordance with the relevant principles of the Declaration of Helsinki.

## 3 Results

As shown in **Figure 1**, among 1,182 patients with rectal cancer who underwent surgery during the study period, 1,045 patients with stage I-III rectal cancer underwent curative resection and were included in the final cohort. Of these, 106 developed recurrence and 939 did not. The median follow-up duration was 21 months (IQR, 9-43). Among patients without recurrence, 397/939 (42.3%) had follow-up of at least 24 months. The distribution of follow-up duration illustrates that a substantial proportion of patients were censored before or around the 24-month time point and that patients treated in later calendar years had shorter available follow-up (**Supplementary Figure S1**). Baseline comparisons showed no statistically significant differences between the recurrence and non-recurrence groups in age, BMI, sex, surgical approach, sphincter preservation rate, TME rate, or neoadjuvant therapy rate. In contrast, significant differences were observed in indicators related to tumor burden and stage (**Table 1**). The recurrence group had a larger maximum tumor diameter, a greater distance from the dentate line, and a higher LNR, as well as higher proportions of T4 disease, N1–2 disease, AJCC stage III, CEA > 5 ng/mL, CA19-9 > 35 U/mL, and adjuvant chemotherapy. The recurrence group also had higher proportions of poor/undifferentiated tumors. Notably, LVI positivity was less frequent among patients with documented recurrence than among those without recurrence, a pattern opposite to the expected adverse direction. This paradoxical distribution was not interpreted as a protective association. Additional year-stratified analyses showed that the proportion of LVI-positive patients increased substantially in later surgery years, when available follow-up was markedly shorter, supporting a calendar-time and follow-up-related explanation for this finding (**Supplementary Table S1**). Observed recurrence rates increased with higher T stage and AJCC stage, with recurrence documented in 18.0% of patients with T4 disease and 18.2% of patients with stage III disease (**Supplementary Table S2**). These values represent observed recurrence proportions under the available follow-up rather than long-term stage-specific recurrence risks.

**Table 1:** Baseline characteristics of the study cohort according to recurrence status.

Variable	No event (n=939)	Recurrence (n=106)	P value
Age of Diagnosis	60.00 [52.00, 67.00]	62.50 [51.00, 69.75]	0.409
BMI	22.00 [20.00, 24.00]	23.00 [21.00, 25.00]	0.139
Tumor Size (cm)	3.00 [3.00, 4.00]	4.00 [3.00, 5.00]	0.013
Distance to Dentate Line (cm)	5.00 [2.50, 9.00]	7.00 [4.00, 10.00]	0.027
Lymph Node Ratio	0.00 [0.00, 0.06]	0.10 [0.00, 0.36]	<0.001
Sex			0.788
Female	363 (38.7)	39 (36.8)	
Male	576 (61.3)	67 (63.2)	
Histology Type			0.187
Adenocarcinoma	864 (92.0)	93 (87.7)	
Mucinous/Signet Ring	75 (8.0)	13 (12.3)	
Tumor Differentiation			<0.001
Well/Moderate	821 (87.4)	79 (74.5)	
Poor/Undifferentiated	118 (12.6)	27 (25.5)	
Lymphovascular Invasion			<0.001
No	552 (58.8)	87 (82.1)	
Yes	387 (41.2)	19 (17.9)	
Surgical Approach			0.585
Open	281 (29.9)	35 (33.0)	
Laparoscopic	658 (70.1)	71 (67.0)	
Sphincter Preservation			0.43
No	245 (26.1)	32 (30.2)	
Yes	694 (73.9)	74 (69.8)	
TME Performed			0.554
No	331 (35.3)	41 (38.7)	
Yes	608 (64.7)	65 (61.3)	
CEA			<0.001
≤5 ng/mL	637 (67.8)	50 (47.2)	
>5 ng/mL	302 (32.2)	56 (52.8)	
CA19-9			0.007
≤35 U/mL	847 (90.2)	86 (81.1)	
>35 U/mL	92 (9.8)	20 (18.9)	
Neoadjuvant Therapy			0.77
No	846 (90.1)	97 (91.5)	
Yes	93 (9.9)	9 (8.5)	
Adjuvant Chemotherapy			<0.001
No	583 (62.1)	38 (35.8)	
Yes	356 (37.9)	68 (64.2)	
T Stage			<0.001
T1-2	334 (35.6)	12 (11.3)	
T3	232 (24.7)	12 (11.3)	
T4	373 (39.7)	82 (77.4)	
N Stage			<0.001
N0	615 (65.5)	34 (32.1)	
N1	229 (24.4)	44 (41.5)	
N2	95 (10.1)	28 (26.4)	
AJCC Stage			<0.001
I	286 (30.5)	10 (9.4)	
II	329 (35.0)	24 (22.6)	
III	324 (34.5)	72 (67.9)	

Continuous variables are presented as median [interquartile range], and categorical variables as number (column percentage). The Wilcoxon rank-sum test was used to compare continuous variables between patients with and without recurrence. Categorical variables were analyzed using Pearson’s chi-square test, with Yates’ continuity correction for 2×2 tables; Fisher’s exact test was adopted for cells with small expected counts. Recurrence status was defined according to time-to-recurrence events during follow-up.

**Figure 2:**
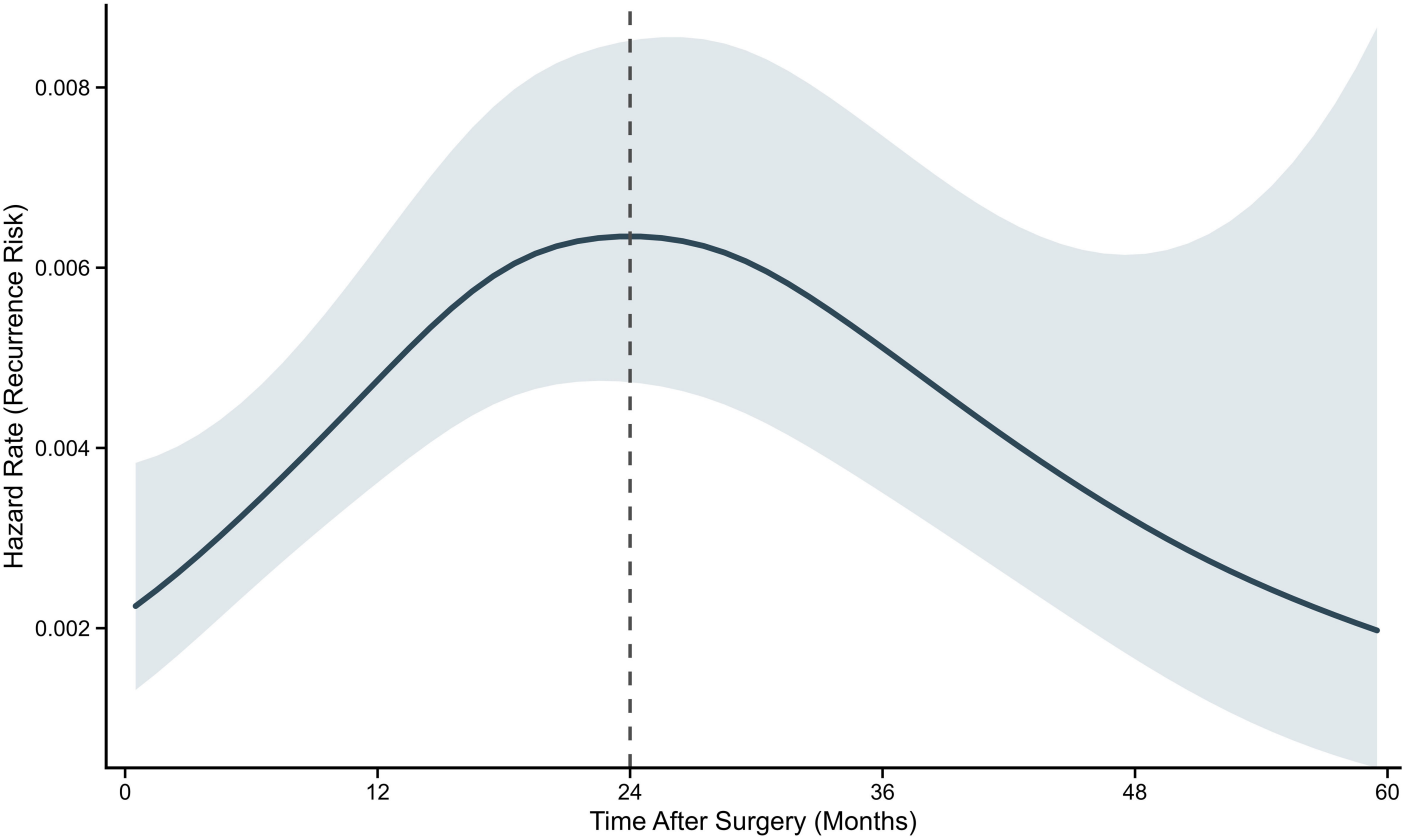
Smoothed hazard curve for time-to-recurrence. Smoothed hazard estimates for time-to-recurrence (TTR) are shown over postoperative follow-up. The recurrence hazard increased after surgery, reached a peak at approximately 24 months, and then gradually declined. This temporal pattern supported the definition of early recurrence as recurrence occurring within 24 months after curative resection.

The smoothed hazard curve showed that postoperative recurrence risk increased during early follow-up and reached its highest estimated level around 24 months after surgery before gradually decreasing thereafter (**Figure 2**). Given the relatively short median follow-up and the width of the confidence band around the estimated peak, the 24-month cutoff was interpreted as a data-informed approximation of an early high-risk window rather than as a precise biological threshold. On the basis of this temporal pattern, recurrence within 24 months after surgery was defined as early recurrence. Because recurrence-free patients with follow-up shorter than 24 months could not be definitively classified as non-early recurrence, the primary logistic analysis was performed in the adequate-follow-up cohort.

In the primary logistic regression analysis using the adequate-follow-up cohort (**Table 2**), univariable analysis showed that multiple indicators related to tumor burden and stage were associated with early recurrence, including poor/undifferentiated differentiation, T stage, N stage, LVI, increased LNR, CEA > 5 ng/mL, CA19-9 > 35 U/mL, and adjuvant chemotherapy. In the multivariable analysis, T4 stage (OR = 2.74, 95% CI 1.13-6.65, P = 0.026) and LNR (per 0.1 increase; OR = 1.36, 95% CI 1.18-1.57, P < 0.001) remained independently associated with early recurrence, whereas CEA >5 ng/mL showed borderline statistical significance (OR = 1.65, 95% CI 0.94-2.88, P = 0.082).

**Table 2:** Univariable and multivariable logistic regression analyses for early recurrence in the adequate-follow-up cohort.

Variable	Univariate analysis			Multivariate analysis		
	OR	95% CI	P value	OR	95% CI	P value
Age of Diagnosis	1.02	0.99-1.04	0.157			
Sex						
Female	Reference					
Male	1.40	0.82-2.41	0.221			
BMI	1.07	0.99-1.15	0.102			
Tumor Size (cm)	1.13	0.98-1.29	0.091			
Distance to Dentate Line (cm)	1.00	0.95-1.06	0.945			
Histology Type						
Adenocarcinoma	Reference					
Mucinous/Signet Ring	1.71	0.78-3.73	0.178			
Tumor Differentiation						
Well/Moderate	Reference			Reference		
Poor/Undifferentiated	2.54	1.38-4.67	0.003	1.19	0.58-2.44	0.644
T Stage						
T1-2	Reference			Reference		
T3	2.92	1.05-8.15	0.041	2.15	0.75-6.20	0.156
T4	5.87	2.60-13.26	<0.001	2.74	1.13-6.65	0.026
N Stage						
N0	Reference					
N1	4.10	2.21-7.60	<0.001			
N2	6.77	3.40-13.47	<0.001			
Lymphovascular Invasion						
No	Reference					
Yes	4.57	2.17-9.63	<0.001			
LNR, per 0.1 increase	1.52	1.34-1.72	<0.001	1.36	1.18-1.57	<0.001
CEA						
≤5 ng/mL	Reference			Reference		
>5 ng/mL	2.48	1.48-4.15	<0.001	1.65	0.94-2.88	0.082
CA19-9						
≤35 U/mL	Reference					
>35 U/mL	2.26	1.16-4.38	0.016			
Neoadjuvant Therapy						
No	Reference					
Yes	1.24	0.50-3.09	0.647			
Adjuvant Chemotherapy						
No	Reference			Reference		
Yes	2.84	1.68-4.82	<0.001	1.39	0.76-2.53	0.281
Surgical Approach						
Open	Reference					
Laparoscopic	1.08	0.63-1.85	0.784			
Sphincter Preservation						
No	Reference					
Yes	0.66	0.38-1.16	0.148			
TME Performed						
No	Reference					
Yes	0.73	0.43-1.24	0.245			

Early recurrence was defined as recurrence occurring within 24 months after curative resection. To reduce outcome misclassification, the primary logistic regression analysis was restricted to patients whose early-recurrence status could be definitively classified, including all patients with documented recurrence and recurrence-free patients with follow-up of at least 24 months. Recurrence-free patients with follow-up shorter than 24 months were excluded from this primary analysis. Lymph node ratio was modeled as a continuous variable; for clinical interpretability, its effect is presented per 0.1 absolute increase.

In the Cox regression analysis for TTR (**Table 3A**), univariable analysis showed that several indicators related to tumor burden and stage were associated with worse TTR. Multivariable analysis showed that T4 stage (HR = 2.86, 95% CI 1.48-5.52, P = 0.002), LNR (per 0.1 increase; HR = 1.28, 95% CI 1.16-1.41, P < 0.001), and CEA > 5 ng/mL (HR = 1.67, 95% CI 1.12-2.50, P = 0.012) were independently associated with TTR. Because death without documented recurrence was treated as censoring in the primary recurrence-focused TTR analysis, we performed a competing-risk sensitivity analysis in which death without documented recurrence was treated as a competing event. Overall, 23 patients died without documented recurrence, including 14 within 24 months and 9 after 24 months. In this sensitivity analysis, T4 stage, higher LNR, and elevated CEA remained associated with a higher cumulative incidence of recurrence, consistent with the primary TTR Cox model (**Supplementary Table S3**).

**Table 3A:** Univariable and multivariable Cox regression analyses for time-to-recurrence.

Variable	Univariate analysis			Multivariate analysis		
	HR	95% CI	P value	HR	95% CI	P value
Age of Diagnosis	1.01	0.99-1.02	0.368			
Sex						
Female	Reference					
Male	1.09	0.73-1.61	0.677			
BMI	1.05	0.99-1.11	0.104			
Tumor Size (cm)	1.12	1.04-1.22	0.004	0.95	0.87-1.05	0.325
Distance to Dentate Line (cm)	1.02	0.97-1.06	0.444			
Histology Type						
Adenocarcinoma	Reference					
Mucinous/Signet Ring	1.55	0.87-2.76	0.141			
Tumor Differentiation						
Well/Moderate	Reference			Reference		
Poor/Undifferentiated	2.47	1.60-3.83	<0.001	1.46	0.90-2.37	0.124
T Stage						
T1-2	Reference			Reference		
T3	1.91	0.86-4.26	0.112	1.42	0.62-3.23	0.403
T4	5.26	2.87-9.64	<0.001	2.86	1.48-5.52	0.002
N Stage						
N0	Reference					
N1	3.73	2.38-5.84	<0.001			
N2	5.12	3.10-8.45	<0.001			
Lymphovascular Invasion						
No	Reference			Reference		
Yes	1.70	0.99-2.92	0.055	0.80	0.45-1.43	0.461
LNR, per 0.1 increase	1.41	1.31-1.52	<0.001	1.28	1.16-1.41	<0.001
CEA						
≤5 ng/mL	Reference			Reference		
>5 ng/mL	2.28	1.56-3.35	<0.001	1.67	1.12-2.50	0.012
CA19-9						
≤35 U/mL	Reference					
>35 U/mL	2.05	1.26-3.33	0.004			
Neoadjuvant Therapy						
No	Reference					
Yes	1.22	0.61-2.41	0.576			
Adjuvant Chemotherapy						
No	Reference			Reference		
Yes	2.86	1.92-4.27	<0.001	1.39	0.89-2.17	0.147
Surgical Approach						
Open	Reference					
Laparoscopic	1.01	0.67-1.52	0.961			
Sphincter Preservation						
No	Reference					
Yes	0.68	0.45-1.02	0.065			
TME Performed						
No	Reference					
Yes	0.78	0.53-1.15	0.212			

Cox proportional hazards models were used to assess associations between clinicopathological variables and time-to-recurrence (TTR). Hazard ratios (HRs) and 95% confidence intervals (CIs) are shown. Variables included in the multivariable model were prespecified according to clinical relevance and collinearity considerations. Lymph node ratio was modeled as a continuous variable; for clinical interpretability, its effect is presented per 0.1 absolute increase.

In the Cox regression analysis for OS (**Table 3B**), univariable analysis showed that multiple clinicopathological factors were associated with worse OS. Multivariable analysis showed that poor/undifferentiated differentiation (HR = 2.66, 95% CI 1.62-4.38, P < 0.001), T4 stage (HR = 2.16, 95% CI 1.10-4.25, P = 0.025), LNR (per 0.1 increase; HR = 1.24, 95% CI 1.11-1.38, P < 0.001), and CEA > 5 ng/mL (HR = 2.16, 95% CI 1.37-3.40, P = 0.001) were independently associated with OS. Testing of the proportional hazards assumption showed no significant overall violation in the TTR model, whereas the overall test for the OS model was borderline, with only tumor differentiation suggesting mild non-proportional hazards (**Supplementary Table S4**). Proportional-hazards sensitivity analyses using stratified Cox and time-varying Cox models showed that estimates for the core variables remained close to those of the primary model, whereas the adverse association of poor/undifferentiated differentiation appeared more pronounced earlier during follow-up and attenuated over time (**Supplementary Tables S5A**, **S5B**; **Supplementary Figure S2**).

**Table 3B:** Univariable and multivariable Cox regression analyses for overall survival.

Variable	Univariate analysis			Multivariate analysis		
	HR	95% CI	P value	HR	95% CI	P value
Age of Diagnosis	1.02	1.00-1.04	0.020			
Sex						
Female	Reference					
Male	1.01	0.65-1.56	0.962			
BMI	1.00	0.93-1.06	0.909			
Tumor Size (cm)	1.17	1.08-1.26	<0.001	0.99	0.91-1.07	0.806
Distance to Dentate Line (cm)	0.98	0.93-1.02	0.318			
Histology Type						
Adenocarcinoma	Reference					
Mucinous/Signet Ring	2.06	1.14-3.72	0.016			
Tumor Differentiation						
Well/Moderate	Reference			Reference		
Poor/Undifferentiated	3.94	2.53-6.15	<0.001	2.66	1.62-4.38	<0.001
T Stage						
T1-2	Reference			Reference		
T3	1.10	0.43-2.79	0.845	0.83	0.32-2.16	0.709
T4	4.06	2.19-7.50	<0.001	2.16	1.10-4.25	0.025
N Stage						
N0	Reference					
N1	3.07	1.85-5.07	<0.001			
N2	5.12	3.01-8.70	<0.001			
Lymphovascular Invasion						
No	Reference			Reference		
Yes	2.50	1.40-4.46	0.002	0.91	0.48-1.75	0.783
LNR, per 0.1 increase	1.39	1.29-1.51	<0.001	1.24	1.11-1.38	<0.001
CEA						
≤5 ng/mL	Reference			Reference		
>5 ng/mL	2.61	1.70-3.99	<0.001	2.16	1.37-3.40	0.001
CA19-9						
≤35 U/mL	Reference					
>35 U/mL	3.30	2.06-5.28	<0.001			
Neoadjuvant Therapy						
No	Reference					
Yes	1.38	0.63-3.00	0.419			
Adjuvant Chemotherapy						
No	Reference			Reference		
Yes	2.13	1.39-3.27	<0.001	0.93	0.56-1.52	0.761
Surgical Approach						
Open	Reference					
Laparoscopic	0.81	0.53-1.26	0.354			
Sphincter Preservation						
No	Reference					
Yes	0.54	0.35-0.85	0.008			
TME Performed						
No	Reference					
Yes	0.72	0.47-1.10	0.132			

Cox proportional hazards models were used to assess associations between clinicopathological variables and overall survival (OS). Hazard ratios (HRs) and 95% confidence intervals (CIs) are shown. Results of proportional hazards testing are provided in the Supplementary Appendix. Mild evidence of non-proportionality for tumor differentiation in the OS model was further addressed in sensitivity analyses. Lymph node ratio was modeled as a continuous variable; for clinical interpretability, its effect is presented per 0.1 absolute increase.

**Figure 3:**
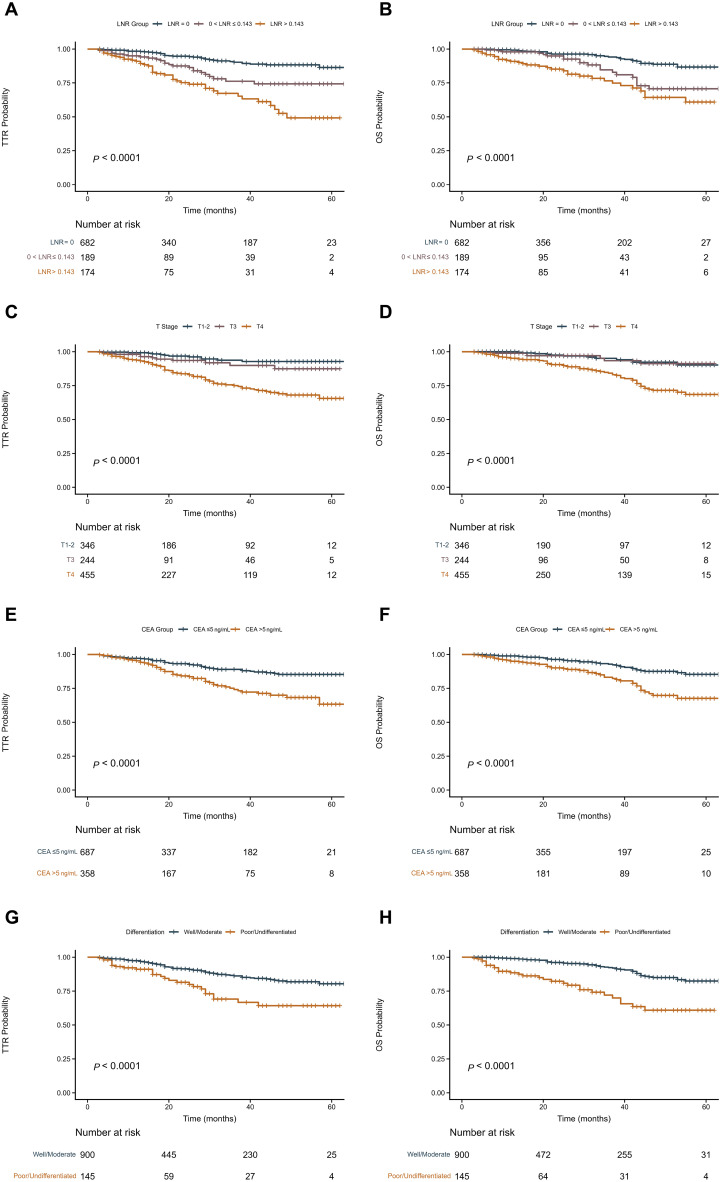
Kaplan-Meier curves for time-to-recurrence and overall survival stratified by lymph node ratio group, T stage, CEA group, and tumor differentiation. Kaplan-Meier curves for time-to-recurrence (TTR) and overall survival (OS) stratified by key clinicopathological variables. Panels are arranged from left to right and from top to bottom as follows: **(A)** TTR stratified by lymph node ratio group; **(B)** OS stratified by lymph node ratio group; **(C)** TTR stratified by T stage; **(D)** OS stratified by T stage; **(E)** TTR stratified by CEA group; **(F)** OS stratified by CEA group; **(G)** TTR stratified by tumor differentiation; **(H)** OS stratified by tumor differentiation. Survival probabilities were estimated using the Kaplan-Meier method and compared using the log-rank test. Numbers at risk are shown below the x-axis.

Kaplan-Meier curves further showed that, after stratification by LNR, T stage, CEA, and tumor differentiation, both TTR and OS were clearly separated, with increased LNR, T4 stage, CEA > 5 ng/mL, and poor/undifferentiated differentiation all associated with worse survival (all P < 0.0001) (**Figure 3**).

**Figure 4:**
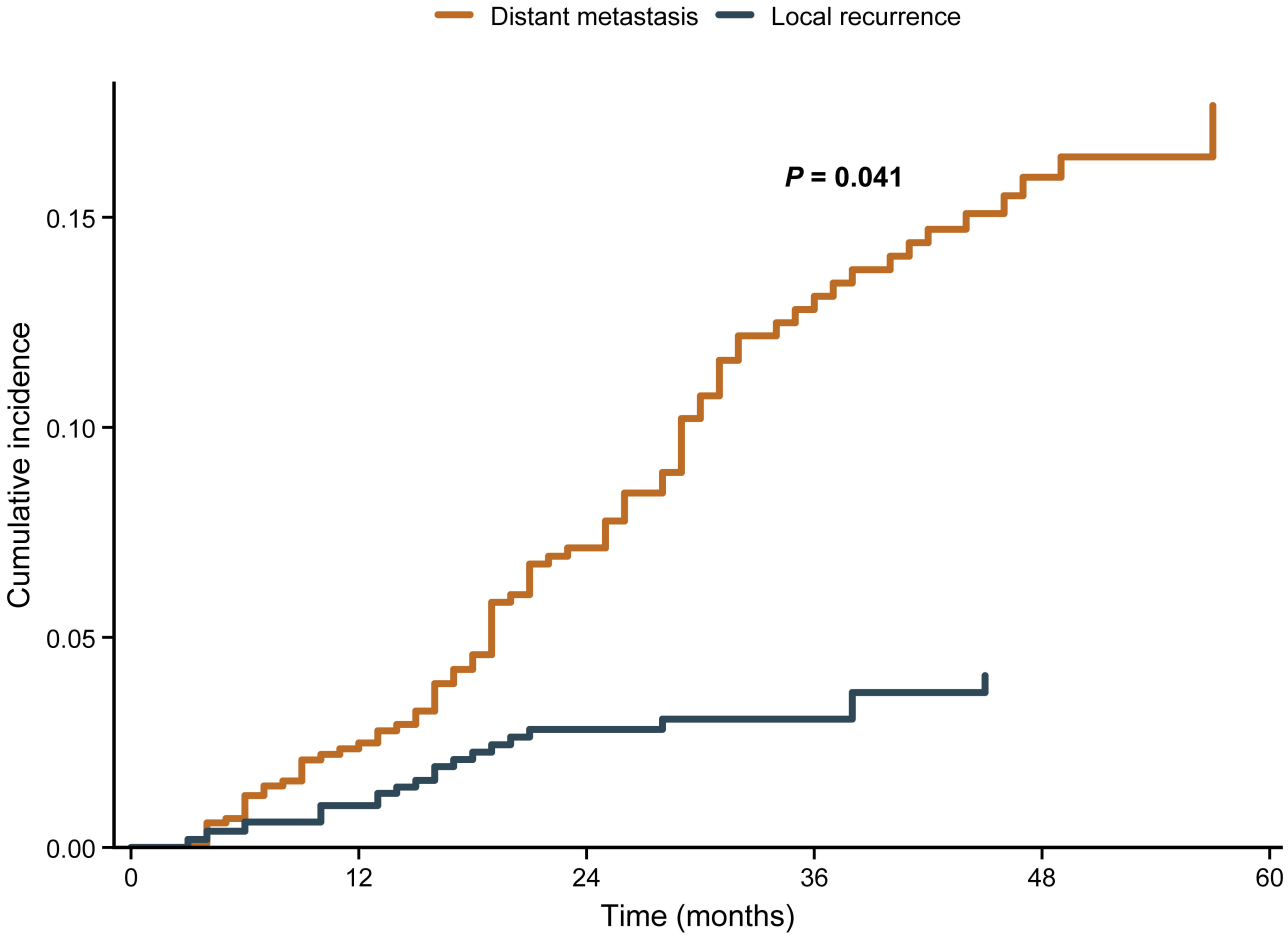
Cumulative incidence curves for recurrence patterns. Cumulative incidence curves showing postoperative recurrence patterns in the overall cohort. Recurrence events were classified as local recurrence and distant metastasis. The curves depict the temporal accumulation of each recurrence pattern after curative resection for stage I-III rectal cancer. The overall numbers at risk at 0, 12, 24, 36, 48, and 60 months were 1,045, 692, 434, 267, 171, and 29, respectively.

**Figure 5:**
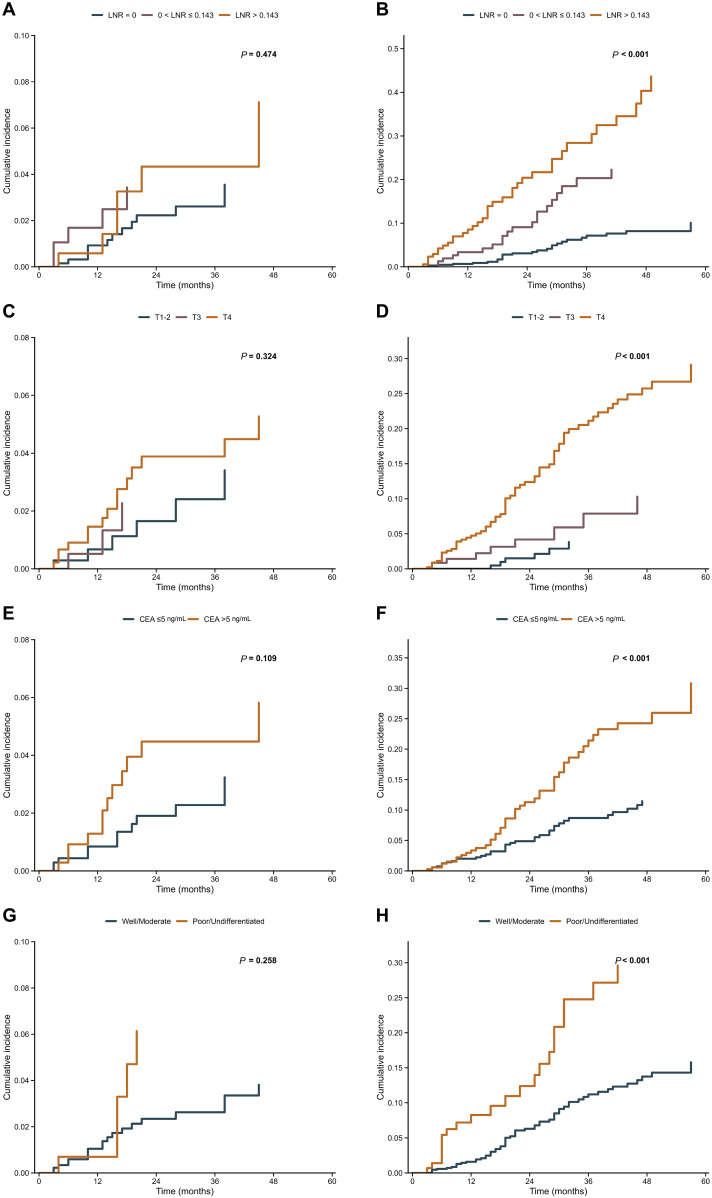
Cumulative incidence curves for recurrence patterns stratified by lymph node ratio group, T stage, CEA group, and tumor differentiation. Cumulative incidence curves for recurrence patterns stratified by key clinicopathological variables. Panels are arranged from left to right and from top to bottom as follows: **(A)** local recurrence stratified by lymph node ratio group; **(B)** distant metastasis stratified by lymph node ratio group; **(C)** local recurrence stratified by T stage; **(D)** distant metastasis stratified by T stage; **(E)** local recurrence stratified by CEA group; **(F)** distant metastasis stratified by CEA group; **(G)** local recurrence stratified by tumor differentiation; **(H)** distant metastasis stratified by tumor differentiation. These analyses illustrate how the major prognostic factors were differentially related to local versus distant failure patterns during follow-up.

The overall cumulative incidence curves for recurrence patterns showed that the cumulative incidence of distant metastasis remained consistently higher than that of local recurrence throughout follow-up. During follow-up, 24 patients developed local recurrence as the first failure event, whereas 82 developed distant metastasis as the first failure event. At approximately 60 months, the cumulative incidence was about 0.17 for distant metastasis and about 0.04 for local recurrence (**Figure 4**). The overall numbers at risk at 0, 12, 24, 36, 48, and 60 months were 1,045, 692, 434, 267, 171, and 29, respectively. Stratified cumulative incidence curves further showed that differences in local recurrence did not reach statistical significance across groups defined by LNR, T stage, CEA, or tumor differentiation, whereas differences in distant metastasis were statistically significant across all of these stratifications (**Figure 5**).

The Fine-Gray competing-risk regression results were consistent with the stratified curves. Because only 24 local recurrence events occurred, the multivariable Fine-Gray model for local recurrence was considered exploratory and underpowered; therefore, the absence of statistically significant correlates should not be interpreted as evidence of no association (**Supplementary Table S6A**). For distant metastasis, univariable analysis showed that multiple indicators related to tumor burden and stage were associated with a higher cumulative incidence; in the multivariable analysis, T4 stage (sHR = 4.22, 95% CI 1.77-10.05, P = 0.001) and LNR (per 0.1 increase; sHR = 1.25, 95% CI 1.13-1.38, P < 0.001) remained statistically significant, whereas CEA > 5 ng/mL showed borderline statistical significance (sHR = 1.57, 95% CI 0.99-2.48, P = 0.055) (**Supplementary Table S6B**).

The full-cohort 24-month logistic model was retained as a sensitivity analysis and yielded results broadly consistent with the primary adequate-follow-up model (**Supplementary Table S7A**). In the extended adequate-follow-up multivariable model including LVI, LNR and T4 stage remained robust, whereas LVI was not independently associated with early recurrence (**Supplementary Table S7B**). In adequate-follow-up alternative-cutoff analyses using 18, 24, and 30 months, LNR remained consistently associated with early recurrence, whereas T4 stage showed a consistent adverse direction and reached or approached statistical significance (**Supplementary Table S8A**). Full-cohort alternative-cutoff analyses were also retained as supplementary sensitivity analyses (**Supplementary Table S8B**).

Internal validation showed acceptable discrimination and overall calibration performance. The primary early-recurrence logistic model had an apparent AUC of 0.760 and an optimism-corrected AUC of 0.740, whereas the apparent and optimism-corrected C-indices were 0.744 and 0.731 for the TTR Cox model and 0.779 and 0.761 for the OS Cox model, respectively. The Brier score of the primary adequate-follow-up early-recurrence logistic model was further contextualized by the corresponding null and scaled Brier scores (**Supplementary Table S9**; **Supplementary Figure S3, S4**). RCS analysis showed statistically significant overall associations of LNR with early recurrence, TTR, and OS, without clear evidence of nonlinearity (**Supplementary Table S10**, **Supplementary Figure S5**). Exploratory decision-curve analyses showed that the multivariable early-recurrence models provided positive net benefit across a clinically plausible range of threshold probabilities in both the adequate-follow-up and full-cohort analyses, although the incremental benefit over LNR alone was modest (**Supplementary Figure S6**).

Supplementary distributional analyses suggested that LVI showed endpoint-dependent behavior rather than a stable independent effect across all models (**Supplementary Table S11**, **Supplementary Figure S7**). Additional supplementary analyses summarized pairwise correlations among candidate predictors and the endpoint-specific model-construction rationale (**Supplementary Tables S12**, **S13**). Baseline comparisons related to the adequate-follow-up restriction are summarized (**Supplementary Tables S14A, S14B**). Several baseline characteristics differed between the retained and excluded groups, most notably LVI; among recurrence-free patients, LVI positivity was substantially more common in the excluded short-follow-up group than in the retained adequate-follow-up group (68.6% vs 3.8%).

## 4 Discussion

The principal finding of this study was that recurrence risk after curative resection for rectal cancer was not evenly distributed throughout follow-up but instead formed a distinct peak at approximately 24 months after surgery. However, because the median follow-up was 21 months and many recurrence-free patients were censored before 24 months, the exact location of the hazard peak should be interpreted cautiously. Around this time window, LNR and T4 stage showed the most stable and consistent associations across early recurrence, TTR, OS, and the risk of distant metastasis. In other words, this study not only identified the high-risk postoperative phase for recurrence but also suggested that regional nodal tumor burden and local invasiveness of the primary tumor constitute the core risk axis linking recurrence timing, long-term outcomes, and failure patterns. Overall, these findings place three clinical questions within a unified analytical framework: when recurrence occurs, who is more likely to recur early, and how recurrence is primarily manifested.

Unlike most previous studies that treated recurrence as a static endpoint, the present study first focused on the temporal distribution of recurrence risk during follow-up. The hazard curve showed that recurrence risk peaked at approximately 24 months after surgery, indicating clear temporal clustering of postoperative recurrence (7, 8). By defining early recurrence according to the hazard function rather than adopting an empirical cutoff directly, the definition used here was more closely aligned with the observed high-risk window in clinical practice (7, 8). Nevertheless, because the 24-month cutoff was derived from the same cohort and the available follow-up was relatively short, it should be interpreted as a data-informed approximation of an early high-risk window rather than a definitive universal cutoff. The broad confidence band around the estimated peak also suggests that 24 months represents one clinically interpretable cutoff within a broader plausible high-risk period rather than a precise biological threshold.

In the primary multivariable analysis of early recurrence, which was restricted to the adequate-follow-up cohort, LNR and T4 stage were the most stable independent correlates. This design was chosen because recurrence-free patients censored before 24 months could not be definitively classified as non-early recurrence. Alternative-cutoff analyses further showed that LNR remained consistently associated with early recurrence, whereas T4 stage showed a consistent adverse direction and reached or approached statistical significance. This suggests that regional nodal tumor burden and local invasiveness of the primary lesion jointly represent the major features of early postoperative failure risk (4, 10, 18, 19). LNR reflects nodal tumor burden, whereas T4 stage indicates more aggressive local invasion (10, 11, 18). By contrast, CEA showed only borderline statistical significance in the early recurrence model, although the direction of association remained generally consistent (4, 20). In addition, the RCS analysis did not show clear nonlinear relationships between LNR and the study endpoints, supporting its use as a continuous burden indicator in the primary models.

An important strength of this study is that the early recurrence analysis did not stand alone but instead formed a relatively complete cross-endpoint chain together with the long-term outcome analyses. Cox regression showed that LNR and T4 stage were associated not only with early recurrence but also independently with worse TTR and OS. CEA remained associated with both TTR and OS, whereas tumor differentiation was mainly reflected at the OS level (10, 11, 21). Kaplan-Meier curves and internal validation further supported this cross-endpoint consistency, suggesting that these core variables are not only statistically associated with outcomes but also have a certain degree of risk-stratification value (4, 11). Because death without documented recurrence was treated as censoring in the recurrence-focused TTR analysis, dependent censoring was a potential concern. However, a competing-risk sensitivity analysis treating death without documented recurrence as a competing event yielded findings consistent with the primary TTR Cox model, supporting the robustness of the main recurrence-focused results.

Not all variables behaved identically across endpoints. CEA did not reach the conventional threshold for statistical significance in the early recurrence model but remained independently associated with both TTR and OS, suggesting that it may be more reflective of overall recurrence and mortality risk (20, 21). Tumor differentiation was mainly evident at the OS level (4, 11). These differences indicate that distinct indicators may capture different dimensions of information related to recurrence timing, overall recurrence risk, and mortality risk, which further supports modeling early recurrence, long-term outcomes, and failure patterns separately rather than collapsing them into a single composite endpoint. The behavior of LVI deserves specific comment. In the full-cohort early-recurrence analysis, LVI showed an apparently protective univariable association, whereas in the adequate-follow-up cohort it showed the expected adverse direction, with the univariable OR changing from 0.34 to 4.57. This reversal is most plausibly explained, at least in part, by outcome misclassification in the full-cohort binary endpoint, in which recurrence-free patients with follow-up shorter than 24 months were classified as non-early recurrence. The baseline comparison further showed that LVI positivity was disproportionately common among excluded short-follow-up recurrence-free patients, supporting the adequate-follow-up framework for the primary early-recurrence analysis. The year-stratified analysis further supported this interpretation, showing that LVI positivity increased markedly in later surgery years, when available follow-up was substantially shorter. Thus, the observed association of LVI was interpreted as endpoint- and follow-up-dependent rather than as a stable determinant comparable to LNR and T4 stage.

The competing-risk analysis further showed that postoperative recurrence in rectal cancer is not a homogeneous event. The overall CIF curves demonstrated that the cumulative incidence of distant metastasis remained consistently higher than that of local recurrence throughout follow-up, and after stratification by LNR, T stage, CEA, and tumor differentiation, between-group separation was more pronounced for distant metastasis. The Fine-Gray regression results were consistent with this pattern: LNR and T4 stage remained independently associated with distant metastasis, whereas the local-recurrence model was underpowered because only 24 local recurrence events occurred. Therefore, the absence of statistically significant predictors for local recurrence should not be interpreted as evidence of no association (11, 22). Although the relatively small number of local recurrence events limited statistical power, the overall findings suggest that the postoperative failure burden in this cohort was driven primarily by distant metastasis (12).

It is noteworthy that tumor differentiation showed only mild non-proportional hazards in the OS model. In the stratified Cox and time-varying Cox models, the estimates for LNR, T4 stage, and CEA remained close to those of the primary model, indicating that the main conclusions were not materially affected. Moreover, the time-varying effect plot suggested that the relative adverse effect of poor/undifferentiated differentiation was more apparent in the early postoperative period and gradually attenuated thereafter.

From a clinical perspective, the value of this study lies in its implications for risk-stratified postoperative surveillance. The 24-month high-risk window identified by the hazard curve suggests that the first two postoperative years may represent the most critical monitoring period. LNR, T4 stage, and selected indicators associated with long-term outcomes may help identify higher-risk patients relatively early after surgery, while the competing-risk analysis further suggests that their major risk burden is more closely related to distant metastasis (23). Accordingly, the present findings support further evaluation of surveillance strategies stratified by pathological burden and invasive features, in which patients with elevated LNR, T4 disease, and unfavorable biological features may require closer monitoring during the first 24 months after surgery, with particular attention to distant metastatic surveillance (6).

The core variables included in this study were all derived from routine pathological or biochemical data, which supports their accessibility and practical applicability. Therefore, even without incorporating complex molecular testing, a preliminary stratification of postoperative recurrence timing risk and failure patterns may still be achievable (1, 6). As postoperative management increasingly moves toward individualized risk assessment and dynamic monitoring, these routine indicators may continue to serve as the most fundamental tools for risk stratification. Future integration of molecular biomarkers, particularly circulating tumor DNA/minimal residual disease (ctDNA/MRD) monitoring, may help build a more dynamic postoperative surveillance framework, and the time-window information and baseline risk-stratification evidence provided by this study may offer relevant clinical context for such efforts (24, 25).

The strengths of this study are several. First, the high-risk window was defined on the basis of the temporal distribution of postoperative recurrence hazard, providing a more data-driven definition of early recurrence. Second, early recurrence, TTR, OS, and competing-risk analysis were integrated within a single framework, allowing the main findings to corroborate one another across endpoints. Third, multiple sensitivity analyses were performed around the key conclusions, including adequate-follow-up and full-cohort early-recurrence analyses, alternative cutoff definitions, proportional-hazards sensitivity analyses, and internal validation, thereby strengthening the robustness of the results. Finally, the study simultaneously considered overall recurrence and recurrence patterns, providing a more complete description of postoperative failure pathways.

This study also has limitations. First, as a single-center retrospective analysis, it cannot fully avoid selection bias and residual confounding. In addition, neoadjuvant therapy was used in a minority of patients during the study period, reflecting the real-world treatment context of our cohort. Therefore, the observed recurrence dynamics and the prognostic role of postoperative pathological variables, particularly LNR and T stage, may not be directly generalizable to contemporary settings with broader use of neoadjuvant chemoradiotherapy or total neoadjuvant therapy. Second, the relatively small number of local recurrence events limited the statistical power for identifying correlates of local recurrence in the competing-risk models and may also have affected the stability of the stratified local recurrence results. Third, the median follow-up duration was relatively short, and a substantial proportion of recurrence-free patients were censored before 24 months. Therefore, the observed recurrence proportion may underestimate the long-term recurrence burden, and the 24-month cutoff may still have been influenced by the sample distribution and hazard-smoothing approach; it should be externally validated before being used as a definitive clinical threshold. In addition, although the adequate-follow-up restriction reduced outcome misclassification for the binary early-recurrence endpoint, it excluded a substantial number of short-follow-up recurrence-free patients and may have introduced follow-up-related selection; therefore, full-cohort sensitivity analyses were retained to assess the robustness of the main findings. Fourth, the variables included in this study were derived mainly from routine clinicopathological data and did not incorporate molecular biomarkers, depth of treatment response, or more granular imaging information. Finally, although exploratory decision-curve analyses were added to assess potential clinical utility, the findings have not yet been externally validated in an independent cohort, and their generalizability and clinical utility require further evaluation in larger, multicenter, and treatment-era-diverse populations before implementation in surveillance planning.

Although the present findings still require further validation in larger samples and independent external cohorts, they suggest that postoperative recurrence risk in rectal cancer is neither homogeneous nor static, but instead characterized by an identifiable time window and a relatively stable pathological risk axis. In particular, the cross-endpoint consistency of LNR and T4 stage across early recurrence, long-term outcomes, and distant metastasis risk suggests that they may serve as core clinical indicators linking postoperative risk stratification with adjustment of surveillance intensity. On this basis, building a more targeted risk-stratified surveillance strategy around the high-risk period within the first 24 months after surgery may be more clinically useful than a uniform follow-up approach.

## 5 Conclusion

In conclusion, this study showed that recurrence risk after curative resection for rectal cancer had clear temporal dynamics and peaked at approximately 24 months after surgery. LNR and T4 stage were the most stable correlates across early recurrence, TTR, OS, and the risk of distant metastasis, whereas CEA and tumor differentiation were more prominently reflected in long-term outcomes. Postoperative failure was dominated by distant metastasis, and the major high-risk factors showed a more evident influence on distant metastatic risk. These findings provide a basis for further development and validation of postoperative risk-stratified surveillance according to time windows and pathological risk burden.

## Data Availability

The data analyzed in this study is subject to the following licenses/restrictions: The data cannot be shared publicly due to privacy and ethical restrictions. Requests to access these datasets should be directed to Yulong He, heyulong@mail.sysu.edu.cn.
