# Supplementary material for: Temporal dynamics and determinants of early recurrence after curative resection for stage I-III rectal cancer: integrated analyses of hazard function, survival, and competing risks
Source: Front Oncol. 2026 Jun 18;16:1850553. doi: 10.3389/fonc.2026.1850553 (PMC13322826; doi:10.3389/fonc.2026.1850553)
Supplement: Supplementary file 1 [file DataSheet1.docx]

**Supplementary Figures**

| 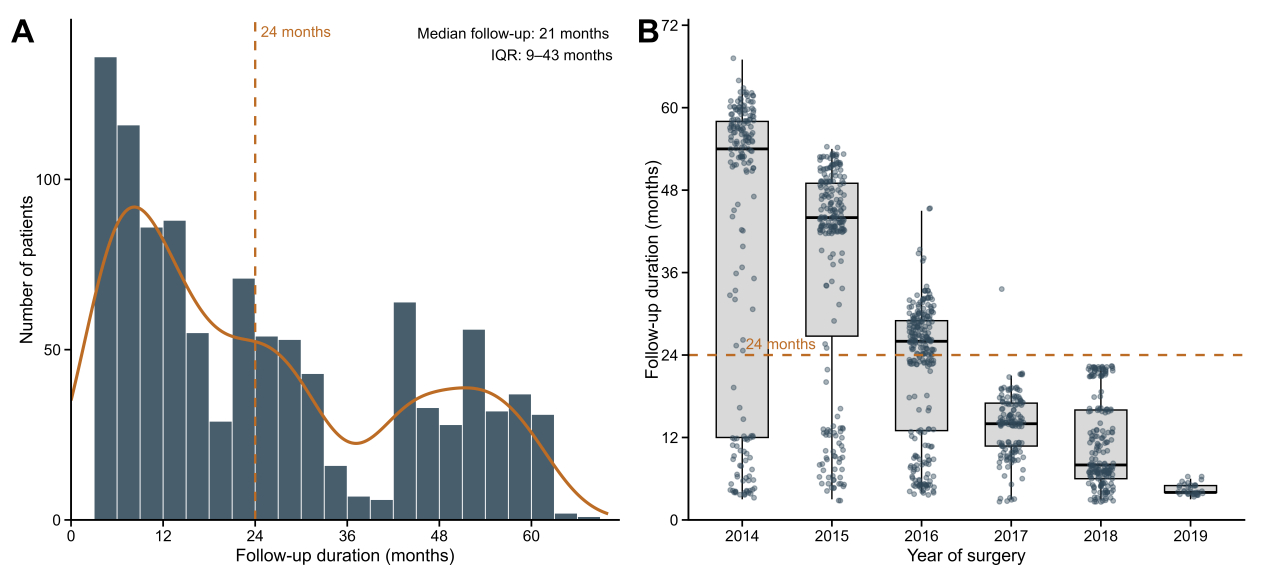 |
| --- |
| **Figure S1. Distribution of follow-up duration in the full cohort and according to year of surgery.** (A) Distribution of follow-up duration in the full cohort. The vertical dashed line indicates 24 months after surgery. (B) Follow-up duration according to year of surgery. The horizontal dashed line indicates 24 months after surgery. Follow-up duration was calculated using overall survival follow-up time. This figure illustrates the censoring pattern underlying the 24-month early-recurrence definition and shows that patients treated in later calendar years had shorter available follow-up. |

| 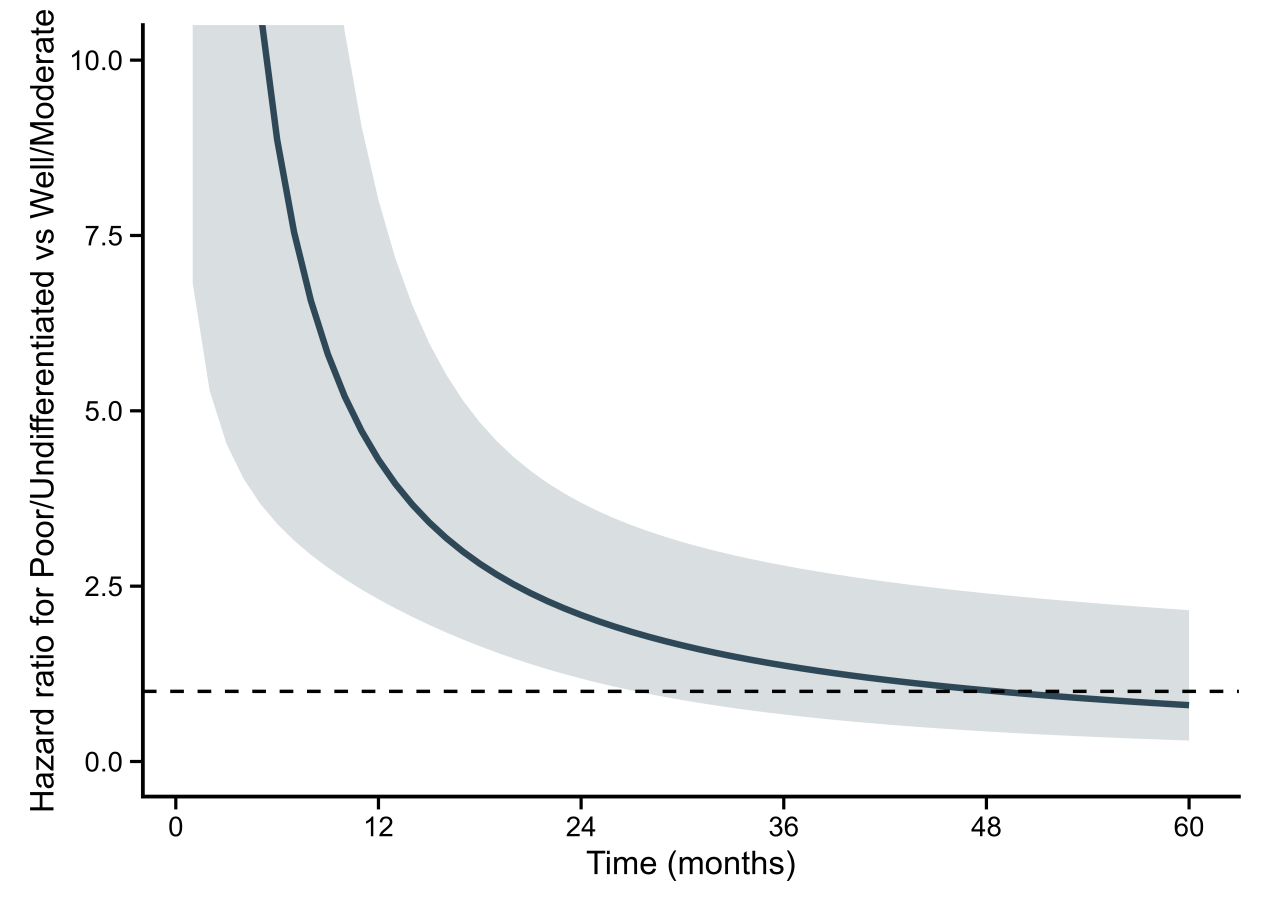 |
| --- |
| **Figure S2. Estimated time-dependent hazard ratio for tumor differentiation in the OS time-varying Cox model.** Estimated time-dependent hazard ratio for poor/undifferentiated versus well/moderate tumor differentiation from the time-varying Cox model for overall survival. The solid line represents the estimated hazard ratio over follow-up time, and the shaded area represents the 95% confidence interval. |

| 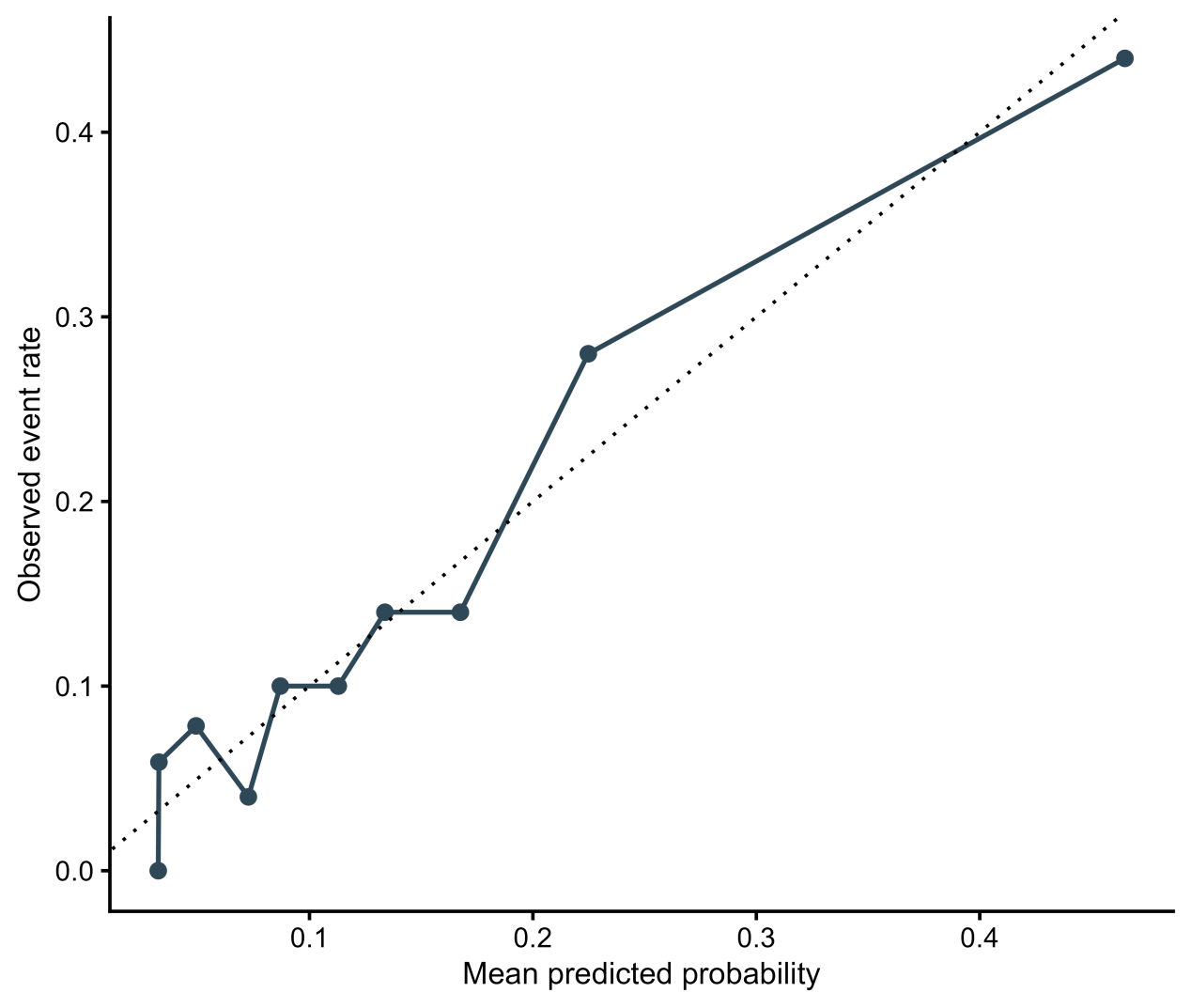 |
| --- |
| **Figure S3. Calibration plot for the primary multivariable logistic regression model of early recurrence in the adequate-follow-up cohort.** Early recurrence was defined as documented recurrence within 24 months after curative resection. The adequate-follow-up cohort included all patients with documented recurrence and recurrence-free patients with follow-up of at least 24 months, whereas recurrence-free patients with follow-up shorter than 24 months were excluded to reduce potential outcome misclassification. The plot shows the agreement between predicted and observed probabilities of early recurrence. The diagonal reference line represents perfect calibration. |

| 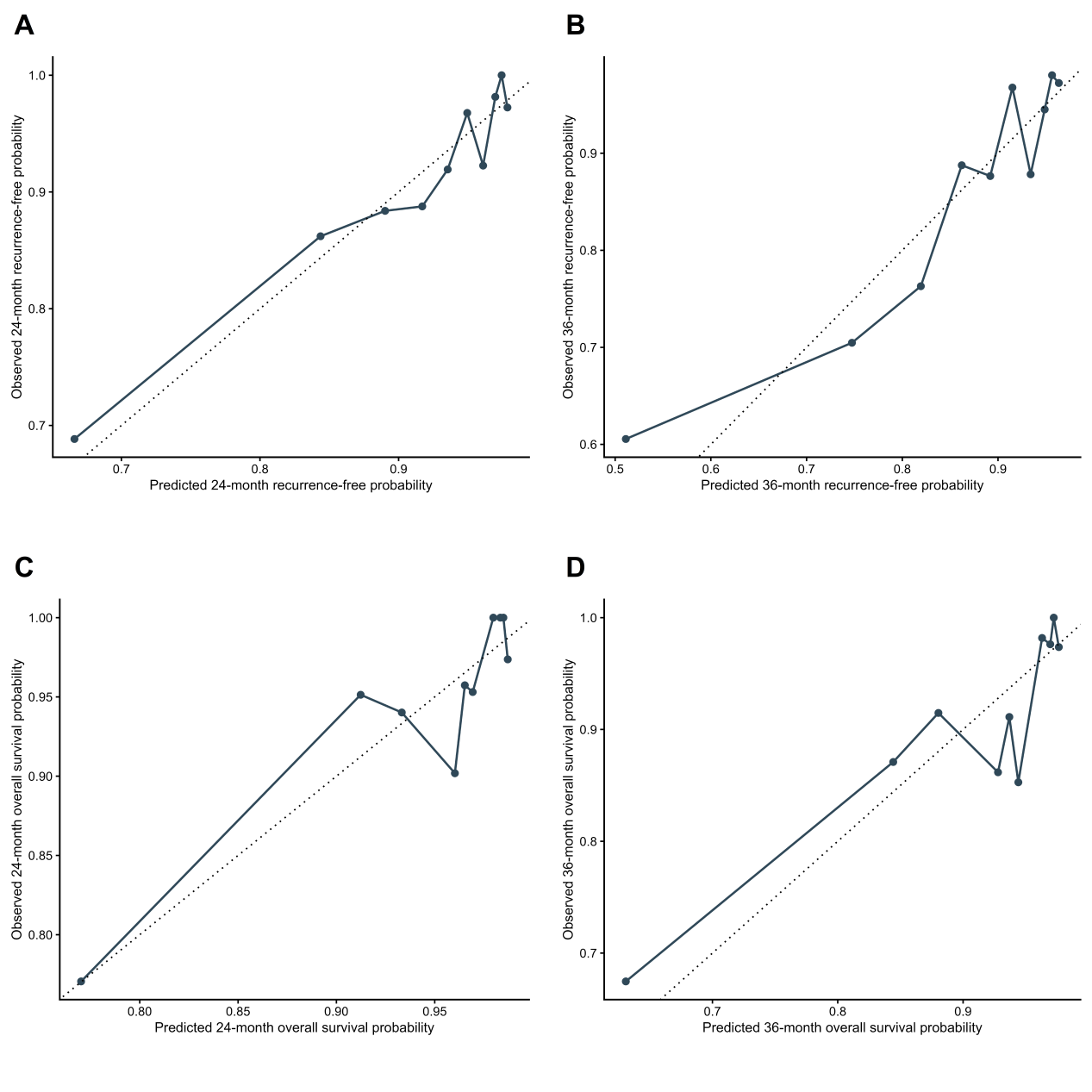 |
| --- |
| **Figure S4. TTR and OS calibration for 24 months and 36 months.** Calibration plots for the multivariable survival models. Panels are arranged from left to right and from top to bottom as follows: (A) TTR calibration at 24 months; (B) TTR calibration at 36 months; (C) OS calibration at 24 months; (D) OS calibration at 36 months. These plots compare predicted and observed survival probabilities at clinically relevant postoperative time points. |

| 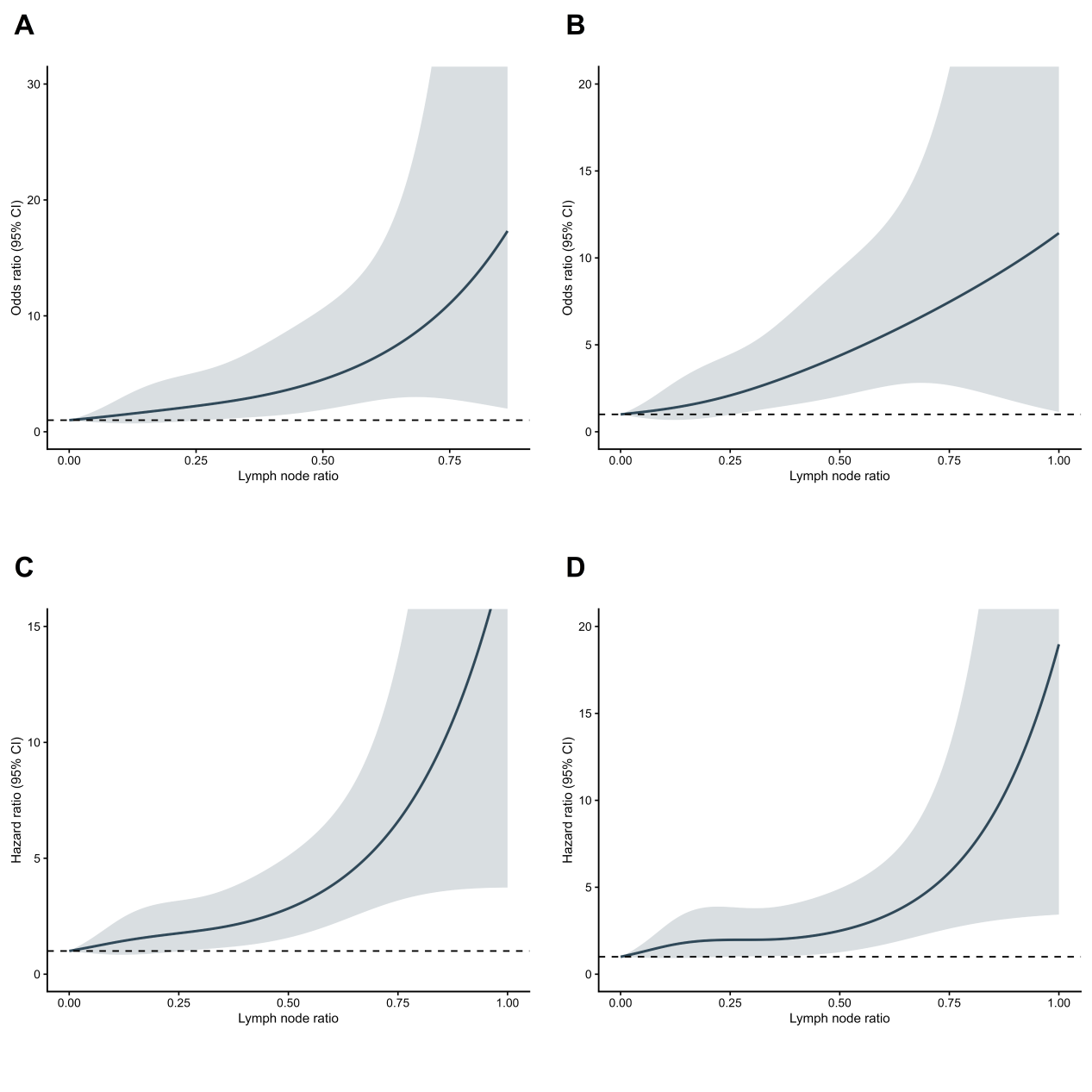 |
| --- |
| ****Figure S5. Restricted cubic spline analyses of lymph node ratio in relation to early recurrence, time-to-recurrence, and overall survival.**** Restricted cubic spline analyses of lymph node ratio (LNR) in relation to the study endpoints. Panels are arranged from left to right and from top to bottom as follows: **(A)** primary multivariable logistic spline model for early recurrence in the adequate-follow-up cohort; **(B)** full-cohort multivariable logistic spline model for early recurrence as a sensitivity analysis; **(C)** multivariable Cox spline model for TTR; **(D)** multivariable Cox spline model for overall survival. Curves are shown as adjusted odds ratios or hazard ratios with 95% confidence intervals according to LNR, using the median LNR as the reference value. LNR was retained on its original 0-1 scale for spline visualization, whereas regression estimates in the main and supplementary tables are presented per 0.1 absolute increase for clinical interpretability. |

| 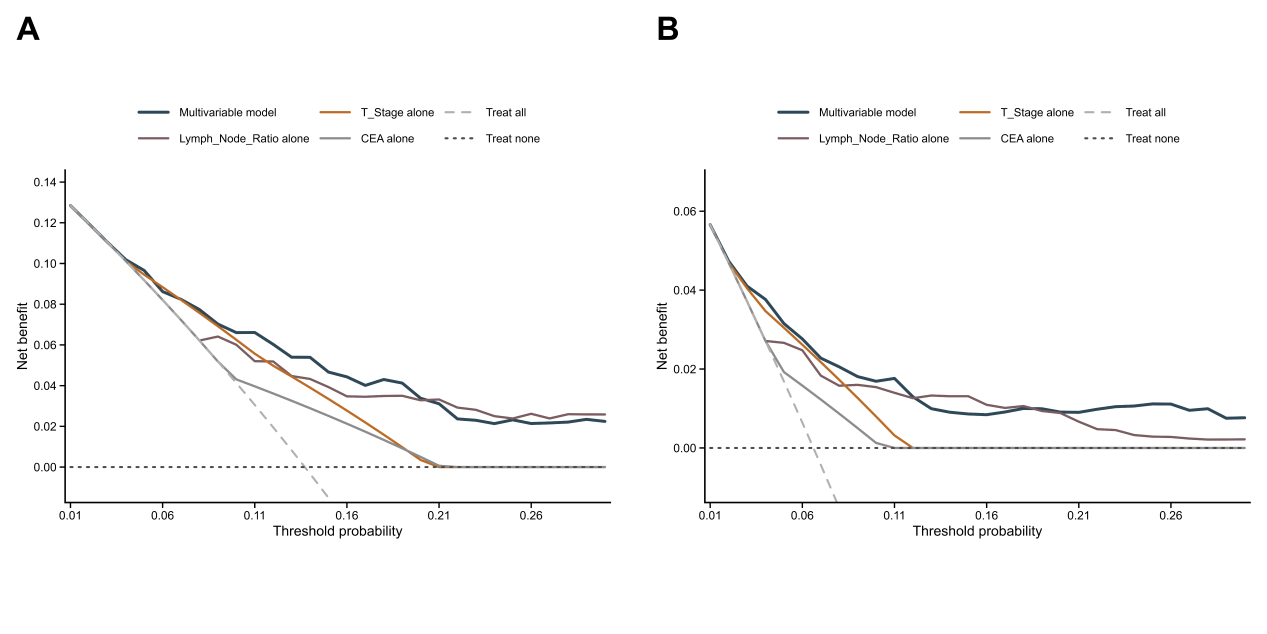 |
| --- |
| **Figure S6. Decision-curve analyses of early-recurrence logistic models.** Decision-curve analyses were performed to evaluate the exploratory clinical utility of the early-recurrence logistic models across threshold probabilities. (**A**) The primary multivariable model fitted in the adequate-follow-up cohort; (**B**) the full-cohort sensitivity model. The multivariable model was compared with single-variable models based on LNR, T stage, and CEA, as well as treat-all and treat-none strategies. Because the two analyses were fitted in different analytic cohorts, the magnitude of net benefit should be interpreted within each panel rather than directly compared across panels. These analyses were exploratory and intended to assess potential clinical utility rather than to establish clinical implementation. |

| 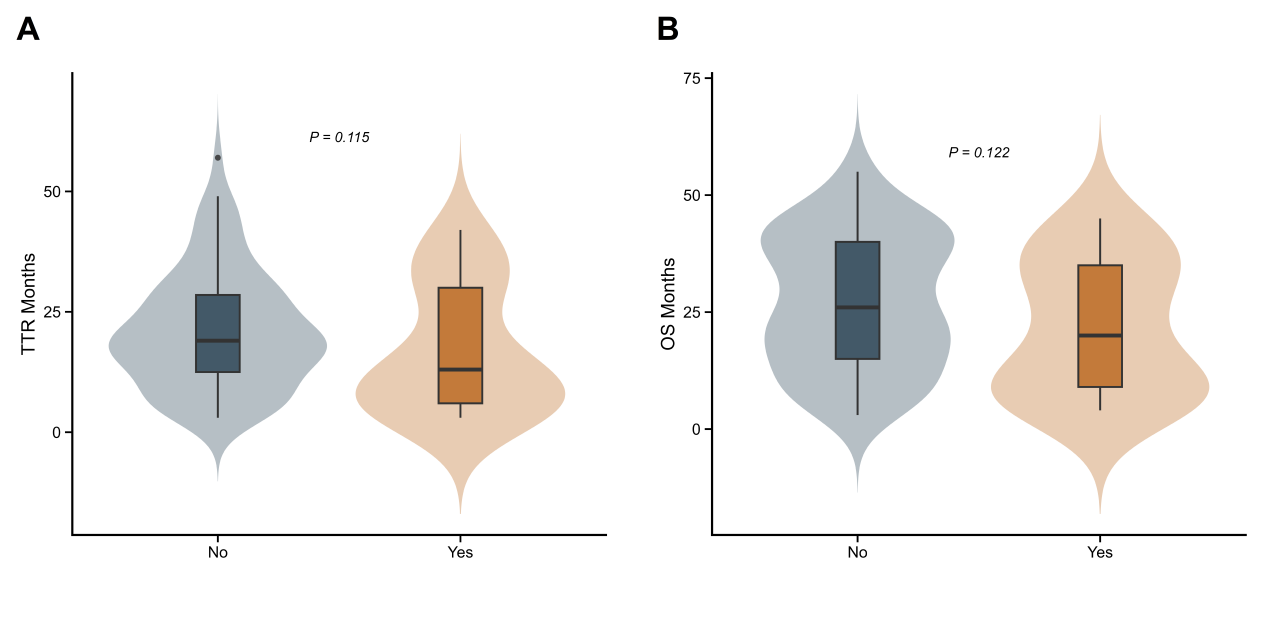 |
| --- |
| **Figure S7. Event-time distributions according to lymphovascular invasion.** Violin-box plots showing event-time distributions according to lymphovascular invasion status. Panels are arranged from left to right and from top to bottom as follows: (**A**) TTR among patients with recurrence events by lymphovascular invasion status; (**B**) overall survival time among patients with death events by lymphovascular invasion status. The box indicates the median and interquartile range, and the violin shape reflects the overall distribution. |
