# Supplementary material for: Temporal dynamics and determinants of early recurrence after curative resection for stage I-III rectal cancer: integrated analyses of hazard function, survival, and competing risks
Source: Front Oncol. 2026 Jun 18;16:1850553. doi: 10.3389/fonc.2026.1850553 (PMC13322826; doi:10.3389/fonc.2026.1850553)
Supplement: Supplementary file 2 [file DataSheet2.docx]

| **Table S1. Distribution of lymphovascular invasion according to year of surgery**   \| Year of surgery \| Total *N* \| LVI negative, *n* (%) \| LVI positive, *n* (%) \| Median follow-up duration, months [IQR] \| Follow-up <24 months, *n* (%) \| Recurrence, *n* (%) \| Early recurrence, *n* (%) \| \| --- \| --- \| --- \| --- \| --- \| --- \| --- \| --- \| \| 2014 \| 192 \| 134 (69.8) \| 58 (30.2) \| 54 [12–58] \| 52 (27.1) \| 26 (13.5) \| 14 (7.3) \| \| 2015 \| 222 \| 158 (71.2) \| 64 (28.8) \| 44 [27–49] \| 54 (24.3) \| 33 (14.9) \| 15 (6.8) \| \| 2016 \| 255 \| 170 (66.7) \| 85 (33.3) \| 26 [13–29] \| 100 (39.2) \| 14 (5.5) \| 7 (2.7) \| \| 2017 \| 144 \| 76 (52.8) \| 68 (47.2) \| 14 [11–17] \| 143 (99.3) \| 12 (8.3) \| 12 (8.3) \| \| 2018 \| 197 \| 95 (48.2) \| 102 (51.8) \| 8 [6–16] \| 197 (100.0) \| 17 (8.6) \| 17 (8.6) \| \| 2019 \| 35 \| 6 (17.1) \| 29 (82.9) \| 4 [4–5] \| 35 (100.0) \| 4 (11.4) \| 4 (11.4) \| \| Overall \| 1045 \| 639 (61.1) \| 406 (38.9) \| 21 [9–43] \| 581 (55.6) \| 106 (10.1) \| 69 (6.6) \| |
| --- | --- | --- | --- | --- | --- | --- | --- | --- | --- | --- | --- | --- | --- | --- | --- | --- | --- | --- | --- | --- | --- | --- | --- | --- | --- | --- | --- | --- | --- | --- | --- | --- | --- | --- | --- | --- | --- | --- | --- | --- | --- | --- | --- | --- | --- | --- | --- | --- | --- | --- | --- | --- | --- | --- | --- | --- | --- | --- | --- | --- | --- | --- | --- | --- |
| Table S1. Lymphovascular invasion was coded as absent or present. Follow-up duration was calculated using overall survival follow-up time and is presented as median [interquartile range]. Follow-up <24 months was defined as available follow-up shorter than 24 months. Recurrence was defined as documented recurrence, and early recurrence was defined as documented recurrence within 24 months after surgery. Percentages were calculated within each year of surgery. Differences in LVI distribution across surgery years were evaluated using the Pearson chi-square test, and the linear trend in LVI-positive proportion across ordered years was evaluated using a trend test. |

| **Table S2. Observed recurrence rates stratified by T stage and AJCC stage**   \| Stratification variable \| Level \| Total *N* \| Recurrence, *n* (%) \| Early recurrence, *n* (%) \| Late recurrence, *n* (%) \| Local recurrence, *n* (%) \| Distant metastasis, *n* (%) \| Median follow-up duration, months [IQR] \| Follow-up ≥24 months, *n* (%) \| \| --- \| --- \| --- \| --- \| --- \| --- \| --- \| --- \| --- \| --- \| \| T stage \| T1-2 \| 346 \| 12 (3.5) \| 7 (2.0) \| 5 (1.4) \| 6 (1.7) \| 6 (1.7) \| 22 [10–43] \| 163 (47.1) \| \| T stage \| T3 \| 244 \| 12 (4.9) \| 9 (3.7) \| 3 (1.2) \| 3 (1.2) \| 9 (3.7) \| 13 [6–28] \| 75 (30.7) \| \| T stage \| T4 \| 455 \| 82 (18.0) \| 53 (11.6) \| 29 (6.4) \| 15 (3.3) \| 67 (14.7) \| 23 [11–44] \| 226 (49.7) \| \| AJCC stage \| Stage I \| 296 \| 10 (3.4) \| 6 (2.0) \| 4 (1.4) \| 5 (1.7) \| 5 (1.7) \| 22 [10–44] \| 140 (47.3) \| \| AJCC stage \| Stage II \| 353 \| 24 (6.8) \| 14 (4.0) \| 10 (2.8) \| 8 (2.3) \| 16 (4.5) \| 22 [8–44] \| 163 (46.2) \| \| AJCC stage \| Stage III \| 396 \| 72 (18.2) \| 49 (12.4) \| 23 (5.8) \| 11 (2.8) \| 61 (15.4) \| 18 [9–32] \| 161 (40.7) \| |
| --- | --- | --- | --- | --- | --- | --- | --- | --- | --- | --- | --- | --- | --- | --- | --- | --- | --- | --- | --- | --- | --- | --- | --- | --- | --- | --- | --- | --- | --- | --- | --- | --- | --- | --- | --- | --- | --- | --- | --- | --- | --- | --- | --- | --- | --- | --- | --- | --- | --- | --- | --- | --- | --- | --- | --- | --- | --- | --- | --- | --- | --- | --- | --- | --- | --- | --- | --- | --- | --- | --- |
| Table S2. Recurrence was defined as documented recurrence during follow-up. Early recurrence was defined as recurrence within 24 months after surgery, and late recurrence as recurrence after 24 months. Local recurrence and distant metastasis were defined according to the first documented failure pattern. Follow-up duration was calculated using overall survival follow-up time and is presented as median [interquartile range]. Percentages were calculated within each T-stage or AJCC-stage category. These values represent observed recurrence proportions under the available follow-up and should not be interpreted as long-term stage-specific recurrence risks. |

| **Table S3. Sensitivity analysis for recurrence treating death without documented recurrence as a competing event**   \| Variable \| Univariate Analysis \| \| \| Multivariate Analysis \| \| \| \| --- \| --- \| --- \| --- \| --- \| --- \| --- \| \| sHR \| 95% CI \| P value \| sHR \| 95% CI \| P value \| \| Age of Diagnosis \| 1.01 \| 0.99-1.02 \| 0.466 \|  \|  \|  \| \| Sex \|  \|  \|  \|  \|  \|  \| \| Female \| Reference \| \| \|  \|  \|  \| \| Male \| 1.09 \| 0.73-1.61 \| 0.676 \|  \|  \|  \| \| BMI \| 1.05 \| 1.00-1.11 \| 0.070 \|  \|  \|  \| \| Tumor Size (cm) \| 1.11 \| 1.06-1.18 \| <0.001 \| 0.95 \| 0.88-1.02 \| 0.144 \| \| Distance to Dentate Line (cm) \| 1.02 \| 0.98-1.06 \| 0.383 \|  \|  \|  \| \| Histology Type \|  \|  \|  \|  \|  \|  \| \| Adenocarcinoma \| Reference \| \| \|  \|  \|  \| \| Mucinous/Signet Ring \| 1.48 \| 0.84-2.63 \| 0.178 \|  \|  \|  \| \| Tumor Differentiation \|  \|  \|  \|  \|  \|  \| \| Well/Moderate \| Reference \| \| \| Reference \| \| \| \| Poor/Undifferentiated \| 2.31 \| 1.49-3.58 \| <0.001 \| 1.37 \| 0.85-2.23 \| 0.199 \| \| T Stage \|  \|  \|  \|  \|  \|  \| \| T1-2 \| Reference \| \| \| Reference \| \| \| \| T3 \| 1.91 \| 0.86-4.25 \| 0.111 \| 1.43 \| 0.64-3.21 \| 0.383 \| \| T4 \| 5.18 \| 2.83-9.47 \| <0.001 \| 2.83 \| 1.49-5.38 \| 0.001 \| \| N Stage \|  \|  \|  \|  \|  \|  \| \| N0 \| Reference \| \| \|  \|  \|  \| \| N1 \| 3.72 \| 2.38-5.80 \| <0.001 \|  \|  \|  \| \| N2 \| 4.92 \| 2.98-8.12 \| <0.001 \|  \|  \|  \| \| Lymphovascular Invasion \|  \|  \|  \|  \|  \|  \| \| No \| Reference \| \| \| Reference \| \| \| \| Yes \| 1.64 \| 0.94-2.84 \| 0.079 \| 0.79 \| 0.43-1.45 \| 0.455 \| \| LNR (per 0.1 increase) \| 1.40 \| 1.31-1.50 \| <0.001 \| 1.27 \| 1.15-1.41 \| <0.001 \| \| CEA \|  \|  \|  \|  \|  \|  \| \| ≤5 ng/mL \| Reference \| \| \| Reference \| \| \| \| >5 ng/mL \| 2.24 \| 1.53-3.28 \| <0.001 \| 1.65 \| 1.10-2.48 \| 0.016 \| \| CA19-9 \|  \|  \|  \|  \|  \|  \| \| ≤35 U/mL \| Reference \| \| \|  \|  \|  \| \| >35 U/mL \| 1.91 \| 1.17-3.10 \| 0.009 \|  \|  \|  \| \| Neoadjuvant Therapy \|  \|  \|  \|  \|  \|  \| \| No \| Reference \| \| \|  \|  \|  \| \| Yes \| 1.19 \| 0.60-2.36 \| 0.610 \|  \|  \|  \| \| Adjuvant Chemotherapy \|  \|  \|  \|  \|  \|  \| \| No \| Reference \| \| \| Reference \| \| \| \| Yes \| 2.87 \| 1.93-4.26 \| <0.001 \| 1.44 \| 0.91-2.29 \| 0.122 \| \| Surgical Approach \|  \|  \|  \|  \|  \|  \| \| Open \| Reference \| \| \|  \|  \|  \| \| Laparoscopic \| 1.01 \| 0.68-1.51 \| 0.953 \|  \|  \|  \| \| Sphincter Preservation \|  \|  \|  \|  \|  \|  \| \| No \| Reference \| \| \|  \|  \|  \| \| Yes \| 0.69 \| 0.45-1.04 \| 0.076 \|  \|  \|  \| \| TME Performed \|  \|  \|  \|  \|  \|  \| \| No \| Reference \| \| \|  \|  \|  \| \| Yes \| 0.78 \| 0.53-1.15 \| 0.208 \|  \|  \|  \| |
| --- | --- | --- | --- | --- | --- | --- | --- | --- | --- | --- | --- | --- | --- | --- | --- | --- | --- | --- | --- | --- | --- | --- | --- | --- | --- | --- | --- | --- | --- | --- | --- | --- | --- | --- | --- | --- | --- | --- | --- | --- | --- | --- | --- | --- | --- | --- | --- | --- | --- | --- | --- | --- | --- | --- | --- | --- | --- | --- | --- | --- | --- | --- | --- | --- | --- | --- | --- | --- | --- | --- | --- | --- | --- | --- | --- | --- | --- | --- | --- | --- | --- | --- | --- | --- | --- | --- | --- | --- | --- | --- | --- | --- | --- | --- | --- | --- | --- | --- | --- | --- | --- | --- | --- | --- | --- | --- | --- | --- | --- | --- | --- | --- | --- | --- | --- | --- | --- | --- | --- | --- | --- | --- | --- | --- | --- | --- | --- | --- | --- | --- | --- | --- | --- | --- | --- | --- | --- | --- | --- | --- | --- | --- | --- | --- | --- | --- | --- | --- | --- | --- | --- | --- | --- | --- | --- | --- | --- | --- | --- | --- | --- | --- | --- | --- | --- | --- | --- | --- | --- | --- | --- | --- | --- | --- | --- | --- | --- | --- | --- | --- | --- | --- | --- | --- | --- | --- | --- | --- | --- | --- | --- | --- | --- | --- | --- | --- | --- | --- | --- | --- | --- | --- | --- | --- | --- | --- | --- | --- | --- | --- | --- | --- | --- | --- | --- | --- | --- | --- | --- | --- | --- | --- | --- | --- | --- | --- | --- | --- | --- | --- | --- | --- | --- | --- | --- | --- | --- | --- | --- | --- | --- | --- | --- | --- | --- | --- | --- | --- | --- | --- | --- | --- | --- | --- | --- | --- | --- | --- | --- | --- | --- | --- | --- | --- | --- | --- | --- | --- | --- | --- | --- | --- | --- | --- | --- | --- | --- | --- | --- | --- | --- | --- | --- | --- | --- | --- | --- | --- | --- | --- | --- | --- | --- | --- | --- | --- | --- | --- | --- | --- | --- | --- | --- | --- | --- | --- | --- | --- | --- | --- | --- | --- | --- | --- | --- | --- | --- | --- | --- | --- | --- | --- | --- | --- | --- | --- | --- | --- | --- | --- | --- | --- | --- | --- | --- |
| Table S3. Recurrence was analyzed using a Fine-Gray subdistribution hazard model, with documented recurrence as the target event and death without documented recurrence as the competing event. Patients alive without documented recurrence were treated as censored. The time variable was defined as time to recurrence for patients with documented recurrence and overall survival follow-up time for patients without documented recurrence. The multivariable model used the same covariate structure as the primary TTR Cox model, including tumor size, tumor differentiation, T stage, lymphovascular invasion, lymph node ratio, CEA, and adjuvant chemotherapy. Lymph node ratio was modeled as a continuous variable and is presented per 0.1 absolute increase. |

| **Table S4. PH assumption test results for TTR and OS Cox models**   \| Variable \| TTR P *value* \| OS P *value* \| \| --- \| --- \| --- \| \| Lymph Node Ratio \| 0.637 \| 0.164 \| \| T Stage \| 0.800 \| 0.790 \| \| Tumor Differentiation \| 0.500 \| 0.013 \| \| Lymphovascular Invasion \| 0.387 \| 0.850 \| \| Tumor Size (cm) \| 0.259 \| 0.797 \| \| CEA \| 0.155 \| 0.785 \| \| Adjuvant Chemotherapy \| 0.360 \| 0.114 \| \| GLOBAL \| 0.541 \| 0.067 \| |
| --- | --- | --- | --- | --- | --- | --- | --- | --- | --- | --- | --- | --- | --- | --- | --- | --- | --- | --- | --- | --- | --- | --- | --- | --- | --- | --- | --- |
| Table S4. Proportional hazards assumptions were assessed using Schoenfeld residual-based tests for the multivariable Cox models of time-to-recurrence and overall survival. P values are shown for each covariate and for the global model test. |

| **Table S5A. Proportional-hazards sensitivity analyses for the multivariable OS Cox model**   \| Model \| Variable \| HR \| 95% CI \| P *value* \| \| --- \| --- \| --- \| --- \| --- \| \| Original Cox \| LNR (per 0.1 increase) \| 1.24 \| 1.11-1.38 \| <0.001 \| \| Original Cox \| T Stage T3 \| 0.83 \| 0.32-2.16 \| 0.709 \| \| Original Cox \| T Stage T4 \| 2.16 \| 1.10-4.25 \| 0.025 \| \| Original Cox \| Lymphovascular Invasion Yes \| 0.91 \| 0.48-1.75 \| 0.783 \| \| Original Cox \| CEA >5 ng/mL \| 2.16 \| 1.37-3.40 \| <0.001 \| \| Original Cox \| Adjuvant Chemotherapy Yes \| 0.93 \| 0.56-1.52 \| 0.761 \| \| Stratified Cox \| LNR (per 0.1 increase) \| 1.23 \| 1.10-1.37 \| <0.001 \| \| Stratified Cox \| T Stage T3 \| 0.82 \| 0.32-2.12 \| 0.679 \| \| Stratified Cox \| T Stage T4 \| 2.13 \| 1.08-4.19 \| 0.029 \| \| Stratified Cox \| Lymphovascular Invasion Yes \| 0.92 \| 0.47-1.79 \| 0.801 \| \| Stratified Cox \| CEA >5 ng/mL \| 2.06 \| 1.31-3.24 \| 0.002 \| \| Stratified Cox \| Adjuvant Chemotherapy Yes \| 0.92 \| 0.56-1.52 \| 0.756 \| \| Time-varying Cox \| LNR (per 0.1 increase) \| 1.23 \| 1.10-1.37 \| <0.001 \| \| Time-varying Cox \| T Stage T3 \| 0.81 \| 0.31-2.10 \| 0.663 \| \| Time-varying Cox \| T Stage T4 \| 2.11 \| 1.07-4.14 \| 0.031 \| \| Time-varying Cox \| Lymphovascular Invasion Yes \| 0.95 \| 0.49-1.83 \| 0.867 \| \| Time-varying Cox \| CEA >5 ng/mL \| 2.03 \| 1.29-3.19 \| 0.002 \| \| Time-varying Cox \| Adjuvant Chemotherapy Yes \| 0.92 \| 0.56-1.52 \| 0.753 \| |
| --- | --- | --- | --- | --- | --- | --- | --- | --- | --- | --- | --- | --- | --- | --- | --- | --- | --- | --- | --- | --- | --- | --- | --- | --- | --- | --- | --- | --- | --- | --- | --- | --- | --- | --- | --- | --- | --- | --- | --- | --- | --- | --- | --- | --- | --- | --- | --- | --- | --- | --- | --- | --- | --- | --- | --- | --- | --- | --- | --- | --- | --- | --- | --- | --- | --- | --- | --- | --- | --- | --- | --- | --- | --- | --- | --- | --- | --- | --- | --- | --- | --- | --- | --- | --- | --- | --- | --- | --- | --- | --- | --- | --- | --- | --- | --- |
| Table S5A. Because tumor differentiation showed mild evidence of non-proportional hazards in the OS model, proportional-hazards sensitivity analyses were performed using alternative model specifications, including a stratified Cox model and a time-varying Cox model. This table summarizes the robustness of core OS predictors across model specifications. Tumor differentiation itself is summarized separately in Table S5B because it was handled differently across the sensitivity models. Lymph node ratio was modeled as a continuous variable; for clinical interpretability, its effect is presented per 0.1 absolute increase. Similar estimates across model specifications support the robustness of the principal OS findings. |

| **Table S5B. Assessment and handling of non-proportional hazards for tumor differentiation in the OS model**   \| **Item** \| **Result or interpretation** \| \| --- \| --- \| \| PH test for tumor differentiation \| Schoenfeld residual-based test *P* = 0.013 \| \| Global PH test for OS model \| *P* = 0.067 \| \| Primary Cox model \| Tumor differentiation was included as an ordinary covariate. Poor/undifferentiated differentiation was associated with worse OS compared with well/moderate differentiation, with HR = 2.66, 95% CI 1.62-4.38, *P* < 0.001. \| \| Stratified Cox sensitivity model \| Tumor differentiation was used as a stratification variable; therefore, its HR was not estimated in this model. The estimates of core variables, including LNR, T stage, and CEA, remained materially unchanged. \| \| Time-varying Cox sensitivity model \| Tumor differentiation was modeled as a time-varying effect. The estimated time-dependent HR is shown in Supplementary Figure S2. \| \| Interpretation \| Tumor differentiation showed mild evidence of non-proportional hazards in the OS model. The adverse association of poor/undifferentiated differentiation appeared more pronounced earlier during follow-up and attenuated over time. Therefore, the primary Cox estimate for tumor differentiation should be interpreted as an average association over the observed follow-up period, whereas the principal findings for LNR, T4 stage, and CEA were robust across model specifications. \| |
| --- | --- | --- | --- | --- | --- | --- | --- | --- | --- | --- | --- | --- | --- | --- |
| Table S5B. This table summarizes the assessment and handling of mild non-proportional hazards for tumor differentiation in the multivariable OS Cox model. PH, proportional hazards; OS, overall survival; HR, hazard ratio; CI, confidence interval; LNR, lymph node ratio; CEA, carcinoembryonic antigen. |

| **Table S6A. Fine-Gray regression analyses for local recurrence**   \| Variable \| Univariate Analysis \| \| \| Multivariate Analysis \| \| \| \| --- \| --- \| --- \| --- \| --- \| --- \| --- \| \| sHR \| 95% CI \| P value \| sHR \| 95% CI \| P value \| \| Age of Diagnosis \| 1.02 \| 0.99-1.06 \| 0.174 \|  \|  \|  \| \| Sex \|  \|  \|  \|  \|  \|  \| \| Female \| Reference \| \| \|  \|  \|  \| \| Male \| 1.26 \| 0.54-2.93 \| 0.595 \|  \|  \|  \| \| BMI \| 1.05 \| 0.95-1.15 \| 0.363 \|  \|  \|  \| \| Tumor Size (cm) \| 0.92 \| 0.73-1.17 \| 0.497 \|  \|  \|  \| \| Distance to Dentate Line (cm) \| 1.00 \| 0.93-1.08 \| 0.902 \|  \|  \|  \| \| Histology Type \|  \|  \|  \|  \|  \|  \| \| Adenocarcinoma \| Reference \| \| \|  \|  \|  \| \| Mucinous/Signet Ring \| 1.52 \| 0.45-5.06 \| 0.498 \|  \|  \|  \| \| Tumor Differentiation \|  \|  \|  \|  \|  \|  \| \| Well/Moderate \| Reference \| \| \| Reference \| \| \| \| Poor/Undifferentiated \| 1.75 \| 0.65-4.68 \| 0.267 \| 1.30 \| 0.49-3.44 \| 0.604 \| \| T Stage \|  \|  \|  \|  \|  \|  \| \| T1-2 \| Reference \| \| \| Reference \| \| \| \| T3 \| 0.90 \| 0.23-3.59 \| 0.880 \| 0.75 \| 0.21-2.71 \| 0.660 \| \| T4 \| 1.80 \| 0.70-4.66 \| 0.225 \| 1.24 \| 0.41-3.72 \| 0.699 \| \| N Stage \|  \|  \|  \|  \|  \|  \| \| N0 \| Reference \| \| \|  \|  \|  \| \| N1 \| 1.34 \| 0.54-3.35 \| 0.527 \|  \|  \|  \| \| N2 \| 1.62 \| 0.53-4.96 \| 0.396 \|  \|  \|  \| \| Lymphovascular Invasion \|  \|  \|  \|  \|  \|  \| \| No \| Reference \| \| \|  \|  \|  \| \| Yes \| 2.63 \| 0.96-7.23 \| 0.061 \|  \|  \|  \| \| LNR (per 0.1 increase) \| 1.20 \| 0.99-1.46 \| 0.065 \| 1.13 \| 0.90-1.41 \| 0.296 \| \| CEA \|  \|  \|  \|  \|  \|  \| \| ≤5 ng/mL \| Reference \| \| \| Reference \| \| \| \| >5 ng/mL \| 1.90 \| 0.85-4.22 \| 0.116 \| 1.65 \| 0.73-3.74 \| 0.227 \| \| CA19-9 \|  \|  \|  \|  \|  \|  \| \| ≤35 U/mL \| Reference \| \| \|  \|  \|  \| \| >35 U/mL \| 2.87 \| 1.13-7.31 \| 0.027 \|  \|  \|  \| \| Neoadjuvant Therapy \|  \|  \|  \|  \|  \|  \| \| No \| Reference \| \| \|  \|  \|  \| \| Yes \| 0.50 \| 0.07-3.69 \| 0.496 \|  \|  \|  \| \| Adjuvant Chemotherapy \|  \|  \|  \|  \|  \|  \| \| No \| Reference \| \| \| Reference \| \| \| \| Yes \| 1.44 \| 0.65-3.20 \| 0.368 \| 1.03 \| 0.41-2.61 \| 0.945 \| \| Surgical Approach \|  \|  \|  \|  \|  \|  \| \| Open \| Reference \| \| \|  \|  \|  \| \| Laparoscopic \| 0.79 \| 0.35-1.79 \| 0.574 \|  \|  \|  \| \| Sphincter Preservation \|  \|  \|  \|  \|  \|  \| \| No \| Reference \| \| \|  \|  \|  \| \| Yes \| 0.74 \| 0.31-1.77 \| 0.497 \|  \|  \|  \| \| TME Performed \|  \|  \|  \|  \|  \|  \| \| No \| Reference \| \| \|  \|  \|  \| \| Yes \| 0.43 \| 0.19-0.95 \| 0.038 \|  \|  \|  \| |
| --- | --- | --- | --- | --- | --- | --- | --- | --- | --- | --- | --- | --- | --- | --- | --- | --- | --- | --- | --- | --- | --- | --- | --- | --- | --- | --- | --- | --- | --- | --- | --- | --- | --- | --- | --- | --- | --- | --- | --- | --- | --- | --- | --- | --- | --- | --- | --- | --- | --- | --- | --- | --- | --- | --- | --- | --- | --- | --- | --- | --- | --- | --- | --- | --- | --- | --- | --- | --- | --- | --- | --- | --- | --- | --- | --- | --- | --- | --- | --- | --- | --- | --- | --- | --- | --- | --- | --- | --- | --- | --- | --- | --- | --- | --- | --- | --- | --- | --- | --- | --- | --- | --- | --- | --- | --- | --- | --- | --- | --- | --- | --- | --- | --- | --- | --- | --- | --- | --- | --- | --- | --- | --- | --- | --- | --- | --- | --- | --- | --- | --- | --- | --- | --- | --- | --- | --- | --- | --- | --- | --- | --- | --- | --- | --- | --- | --- | --- | --- | --- | --- | --- | --- | --- | --- | --- | --- | --- | --- | --- | --- | --- | --- | --- | --- | --- | --- | --- | --- | --- | --- | --- | --- | --- | --- | --- | --- | --- | --- | --- | --- | --- | --- | --- | --- | --- | --- | --- | --- | --- | --- | --- | --- | --- | --- | --- | --- | --- | --- | --- | --- | --- | --- | --- | --- | --- | --- | --- | --- | --- | --- | --- | --- | --- | --- | --- | --- | --- | --- | --- | --- | --- | --- | --- | --- | --- | --- | --- | --- | --- | --- | --- | --- | --- | --- | --- | --- | --- | --- | --- | --- | --- | --- | --- | --- | --- | --- | --- | --- | --- | --- | --- | --- | --- | --- | --- | --- | --- | --- | --- | --- | --- | --- | --- | --- | --- | --- | --- | --- | --- | --- | --- | --- | --- | --- | --- | --- | --- | --- | --- | --- | --- | --- | --- | --- | --- | --- | --- | --- | --- | --- | --- | --- | --- | --- | --- | --- | --- | --- | --- | --- | --- | --- | --- | --- | --- | --- | --- | --- | --- | --- | --- | --- | --- | --- | --- | --- | --- | --- | --- | --- | --- | --- | --- | --- | --- | --- | --- | --- | --- | --- | --- | --- | --- | --- | --- |
| Table S6A. Fine-Gray subdistribution hazard models were used to evaluate associations between clinicopathological variables and the cumulative incidence of local recurrence. Subdistribution hazard ratios (SHRs) and 95% confidence intervals (CIs) are presented. Lymph node ratio was modeled as a continuous variable; for clinical interpretability, its effect is presented per 0.1 absolute increase. Variables included in the multivariable model were prespecified core covariates selected according to the main regression framework. In the multivariable analysis, none of the included variables reached statistical significance, likely reflecting the limited number of local recurrence events and reduced statistical power. |

| **Table S6B. Fine-Gray regression analyses for distant metastasis**   \| Variable \| Univariate Analysis \| \| \| Multivariate Analysis \| \| \| \| --- \| --- \| --- \| --- \| --- \| --- \| --- \| \| sHR \| 95% CI \| P *value* \| sHR \| 95% CI \| P *value* \| \| Age of Diagnosis \| 1.00 \| 0.98-1.02 \| 0.827 \|  \|  \|  \| \| Sex \|  \|  \|  \|  \|  \|  \| \| Female \| Reference \| \| \|  \|  \|  \| \| Male \| 1.04 \| 0.67-1.62 \| 0.873 \|  \|  \|  \| \| BMI \| 1.05 \| 0.99-1.12 \| 0.135 \|  \|  \|  \| \| Tumor Size (cm) \| 1.16 \| 1.09-1.22 \| <0.001 \|  \|  \|  \| \| Distance to Dentate Line (cm) \| 1.02 \| 0.97-1.07 \| 0.436 \|  \|  \|  \| \| Histology Type \|  \|  \|  \|  \|  \|  \| \| Adenocarcinoma \| Reference \| \| \|  \|  \|  \| \| Mucinous/Signet Ring \| 1.52 \| 0.79-2.92 \| 0.208 \|  \|  \|  \| \| Tumor Differentiation \|  \|  \|  \|  \|  \|  \| \| Well/Moderate \| Reference \| \| \|  \|  \|  \| \| Poor/Undifferentiated \| 2.58 \| 1.58-4.21 \| <0.001 \| 1.27 \| 0.75-2.16 \| 0.376 \| \| T Stage \|  \|  \|  \|  \|  \|  \| \| T1-2 \| Reference \| \| \| Reference \| \| \| \| T3 \| 2.89 \| 1.03-8.08 \| 0.043 \| 2.02 \| 0.71-5.76 \| 0.190 \| \| T4 \| 8.42 \| 3.65-19.39 \| <0.001 \| 4.22 \| 1.77-10.05 \| 0.001 \| \| N Stage \|  \|  \|  \|  \|  \|  \| \| N0 \| Reference \| \| \|  \|  \|  \| \| N1 \| 5.09 \| 3.00-8.66 \| <0.001 \|  \|  \|  \| \| N2 \| 7.00 \| 3.90-12.58 \| <0.001 \|  \|  \|  \| \| Lymphovascular Invasion \|  \|  \|  \|  \|  \|  \| \| No \| Reference \| \| \|  \|  \|  \| \| Yes \| 1.30 \| 0.66-2.56 \| 0.441 \|  \|  \|  \| \| LNR (per 0.1 increase) \| 1.42 \| 1.31-1.54 \| <0.001 \| 1.25 \| 1.13-1.38 \| <0.001 \| \| CEA \|  \|  \|  \|  \|  \|  \| \| ≤5 ng/mL \| Reference \| \| \| Reference \| \| \| \| >5 ng/mL \| 2.33 \| 1.51-3.59 \| <0.001 \| 1.57 \| 0.99-2.48 \| 0.055 \| \| CA19-9 \|  \|  \|  \|  \|  \|  \| \| ≤35 U/mL \| Reference \| \| \|  \|  \|  \| \| >35 U/mL \| 1.72 \| 0.98-3.04 \| 0.060 \|  \|  \|  \| \| Neoadjuvant Therapy \|  \|  \|  \|  \|  \|  \| \| No \| Reference \| \| \|  \|  \|  \| \| Yes \| 1.47 \| 0.70-3.05 \| 0.306 \|  \|  \|  \| \| Adjuvant Chemotherapy \|  \|  \|  \|  \|  \|  \| \| No \| Reference \| \| \|  \|  \|  \| \| Yes \| 3.43 \| 2.16-5.45 \| <0.001 \| 1.58 \| 0.92-2.70 \| 0.094 \| \| Surgical Approach \|  \|  \|  \|  \|  \|  \| \| Open \| Reference \| \| \|  \|  \|  \| \| Laparoscopic \| 1.09 \| 0.68-1.73 \| 0.730 \|  \|  \|  \| \| Sphincter Preservation \|  \|  \|  \|  \|  \|  \| \| No \| Reference \| \| \|  \|  \|  \| \| Yes \| 0.68 \| 0.42-1.08 \| 0.099 \|  \|  \|  \| \| TME Performed \|  \|  \|  \|  \|  \|  \| \| No \| Reference \| \| \|  \|  \|  \| \| Yes \| 0.97 \| 0.61-1.52 \| 0.878 \|  \|  \|  \| |
| --- | --- | --- | --- | --- | --- | --- | --- | --- | --- | --- | --- | --- | --- | --- | --- | --- | --- | --- | --- | --- | --- | --- | --- | --- | --- | --- | --- | --- | --- | --- | --- | --- | --- | --- | --- | --- | --- | --- | --- | --- | --- | --- | --- | --- | --- | --- | --- | --- | --- | --- | --- | --- | --- | --- | --- | --- | --- | --- | --- | --- | --- | --- | --- | --- | --- | --- | --- | --- | --- | --- | --- | --- | --- | --- | --- | --- | --- | --- | --- | --- | --- | --- | --- | --- | --- | --- | --- | --- | --- | --- | --- | --- | --- | --- | --- | --- | --- | --- | --- | --- | --- | --- | --- | --- | --- | --- | --- | --- | --- | --- | --- | --- | --- | --- | --- | --- | --- | --- | --- | --- | --- | --- | --- | --- | --- | --- | --- | --- | --- | --- | --- | --- | --- | --- | --- | --- | --- | --- | --- | --- | --- | --- | --- | --- | --- | --- | --- | --- | --- | --- | --- | --- | --- | --- | --- | --- | --- | --- | --- | --- | --- | --- | --- | --- | --- | --- | --- | --- | --- | --- | --- | --- | --- | --- | --- | --- | --- | --- | --- | --- | --- | --- | --- | --- | --- | --- | --- | --- | --- | --- | --- | --- | --- | --- | --- | --- | --- | --- | --- | --- | --- | --- | --- | --- | --- | --- | --- | --- | --- | --- | --- | --- | --- | --- | --- | --- | --- | --- | --- | --- | --- | --- | --- | --- | --- | --- | --- | --- | --- | --- | --- | --- | --- | --- | --- | --- | --- | --- | --- | --- | --- | --- | --- | --- | --- | --- | --- | --- | --- | --- | --- | --- | --- | --- | --- | --- | --- | --- | --- | --- | --- | --- | --- | --- | --- | --- | --- | --- | --- | --- | --- | --- | --- | --- | --- | --- | --- | --- | --- | --- | --- | --- | --- | --- | --- | --- | --- | --- | --- | --- | --- | --- | --- | --- | --- | --- | --- | --- | --- | --- | --- | --- | --- | --- | --- | --- | --- | --- | --- | --- | --- | --- | --- | --- | --- | --- | --- | --- | --- | --- | --- | --- | --- | --- | --- | --- | --- | --- | --- | --- | --- | --- | --- | --- | --- |
| Table S6B. Fine-Gray subdistribution hazard models were used to evaluate associations between clinicopathological variables and the cumulative incidence of distant metastasis. Subdistribution hazard ratios (SHRs) and 95% confidence intervals (CIs) are presented. Lymph node ratio was modeled as a continuous variable; for clinical interpretability, its effect is presented per 0.1 absolute increase. In the multivariable analysis, higher lymph node ratio and T4 stage were independently associated with an increased cumulative incidence of distant metastasis, whereas elevated CEA showed borderline significance. |

| **Table S7A. Sensitivity analysis of early recurrence logistic regression in the full cohort**   \| Variable \| Univariate Analysis \| \| \| Multivariate Analysis \| \| \| \| --- \| --- \| --- \| --- \| --- \| --- \| --- \| \| OR \| 95% CI \| P *value* \| OR \| 95% CI \| P *value* \| \| Age of Diagnosis \| 1.02 \| 0.99-1.04 \| 0.157 \|  \|  \|  \| \| Sex \|  \|  \|  \|  \|  \|  \| \| Female \| Reference \| \| \|  \|  \|  \| \| Male \| 1.36 \| 0.81-2.30 \| 0.246 \|  \|  \|  \| \| BMI \| 1.06 \| 0.98-1.14 \| 0.155 \|  \|  \|  \| \| Tumor Size (cm) \| 1.13 \| 0.99-1.28 \| 0.077 \|  \|  \|  \| \| Distance to Dentate Line (cm) \| 1.04 \| 0.99-1.10 \| 0.142 \|  \|  \|  \| \| Histology Type \|  \|  \|  \|  \|  \|  \| \| Adenocarcinoma \| Reference \| \| \|  \|  \|  \| \| Mucinous/Signet Ring \| 1.70 \| 0.81-3.56 \| 0.157 \|  \|  \|  \| \| Tumor Differentiation \|  \|  \|  \|  \|  \|  \| \| Well/Moderate \| Reference \| \| \| Reference \| \| \| \| Poor/Undifferentiated \| 2.36 \| 1.34-4.17 \| 0.003 \| 1.12 \| 0.59-2.13 \| 0.733 \| \| T Stage \|  \|  \|  \|  \|  \|  \| \| T1-2 \| Reference \| \| \| Reference \| \| \| \| T3 \| 1.85 \| 0.68-5.05 \| 0.227 \| 1.34 \| 0.48-3.73 \| 0.572 \| \| T4 \| 6.38 \| 2.87-14.23 \| <0.001 \| 3.29 \| 1.40-7.74 \| 0.006 \| \| N Stage \|  \|  \|  \|  \|  \|  \| \| N0 \| Reference \| \| \|  \|  \|  \| \| N1 \| 3.59 \| 1.99-6.50 \| <0.001 \|  \|  \|  \| \| N2 \| 6.47 \| 3.39-12.37 \| <0.001 \|  \|  \|  \| \| Lymphovascular Invasion \|  \|  \|  \|  \|  \|  \| \| No \| Reference \| \| \|  \|  \|  \| \| Yes \| 0.34 \| 0.19-0.64 \| <0.001 \|  \|  \|  \| \| LNR (per 0.1 increase) \| 1.49 \| 1.34-1.65 \| <0.001 \| 1.32 \| 1.17-1.49 \| <0.001 \| \| CEA \|  \|  \|  \|  \|  \|  \| \| ≤5 ng/mL \| Reference \| \| \| Reference \| \| \| \| >5 ng/mL \| 2.36 \| 1.44-3.86 \| <0.001 \| 1.56 \| 0.93-2.64 \| 0.094 \| \| CA19-9 \|  \|  \|  \|  \|  \|  \| \| ≤35 U/mL \| Reference \| \| \|  \|  \|  \| \| >35 U/mL \| 2.28 \| 1.22-4.25 \| 0.009 \|  \|  \|  \| \| Neoadjuvant Therapy \|  \|  \|  \|  \|  \|  \| \| No \| Reference \| \| \|  \|  \|  \| \| Yes \| 0.87 \| 0.37-2.07 \| 0.758 \|  \|  \|  \| \| Adjuvant Chemotherapy \|  \|  \|  \|  \|  \|  \| \| No \| Reference \| \| \| Reference \| \| \| \| Yes \| 2.76 \| 1.66-4.59 \| <0.001 \| 1.39 \| 0.80-2.43 \| 0.243 \| \| Surgical Approach \|  \|  \|  \|  \|  \|  \| \| Open \| Reference \| \| \|  \|  \|  \| \| Laparoscopic \| 0.86 \| 0.51-1.44 \| 0.563 \|  \|  \|  \| \| Sphincter Preservation \|  \|  \|  \|  \|  \|  \| \| No \| Reference \| \| \|  \|  \|  \| \| Yes \| 0.76 \| 0.45-1.28 \| 0.296 \|  \|  \|  \| \| TME Performed \|  \|  \|  \|  \|  \|  \| \| No \| Reference \| \| \|  \|  \|  \| \| Yes \| 0.85 \| 0.52-1.40 \| 0.526 \|  \|  \|  \| |
| --- | --- | --- | --- | --- | --- | --- | --- | --- | --- | --- | --- | --- | --- | --- | --- | --- | --- | --- | --- | --- | --- | --- | --- | --- | --- | --- | --- | --- | --- | --- | --- | --- | --- | --- | --- | --- | --- | --- | --- | --- | --- | --- | --- | --- | --- | --- | --- | --- | --- | --- | --- | --- | --- | --- | --- | --- | --- | --- | --- | --- | --- | --- | --- | --- | --- | --- | --- | --- | --- | --- | --- | --- | --- | --- | --- | --- | --- | --- | --- | --- | --- | --- | --- | --- | --- | --- | --- | --- | --- | --- | --- | --- | --- | --- | --- | --- | --- | --- | --- | --- | --- | --- | --- | --- | --- | --- | --- | --- | --- | --- | --- | --- | --- | --- | --- | --- | --- | --- | --- | --- | --- | --- | --- | --- | --- | --- | --- | --- | --- | --- | --- | --- | --- | --- | --- | --- | --- | --- | --- | --- | --- | --- | --- | --- | --- | --- | --- | --- | --- | --- | --- | --- | --- | --- | --- | --- | --- | --- | --- | --- | --- | --- | --- | --- | --- | --- | --- | --- | --- | --- | --- | --- | --- | --- | --- | --- | --- | --- | --- | --- | --- | --- | --- | --- | --- | --- | --- | --- | --- | --- | --- | --- | --- | --- | --- | --- | --- | --- | --- | --- | --- | --- | --- | --- | --- | --- | --- | --- | --- | --- | --- | --- | --- | --- | --- | --- | --- | --- | --- | --- | --- | --- | --- | --- | --- | --- | --- | --- | --- | --- | --- | --- | --- | --- | --- | --- | --- | --- | --- | --- | --- | --- | --- | --- | --- | --- | --- | --- | --- | --- | --- | --- | --- | --- | --- | --- | --- | --- | --- | --- | --- | --- | --- | --- | --- | --- | --- | --- | --- | --- | --- | --- | --- | --- | --- | --- | --- | --- | --- | --- | --- | --- | --- | --- | --- | --- | --- | --- | --- | --- | --- | --- | --- | --- | --- | --- | --- | --- | --- | --- | --- | --- | --- | --- | --- | --- | --- | --- | --- | --- | --- | --- | --- | --- | --- | --- | --- | --- | --- | --- | --- | --- | --- | --- | --- | --- | --- | --- | --- | --- | --- | --- | --- | --- | --- |
| Table S7A. In this sensitivity analysis, all 1,045 patients were included. Patients without documented recurrence were classified as non-early recurrence regardless of follow-up duration. Because some recurrence-free patients had follow-up shorter than 24 months, this analysis was considered supplementary. Lymph node ratio was modeled as a continuous variable; for clinical interpretability, its effect is presented per 0.1 absolute increase. |

| **Table S7B. Extended multivariable logistic regression model for early recurrence including lymphovascular invasion**   \| Variable \| Adjusted OR \| 95% CI \| *P value* \| \| --- \| --- \| --- \| --- \| \| Tumor Differentiation \|  \|  \|  \| \| Well/Moderate \| Reference \| \| \| \| Poor/Undifferentiated \| 1.09 \| 0.52-2.30 \| 0.813 \| \| T Stage \|  \|  \|  \| \| T1-2 \| Reference \| \| \| \| T3 \| 2.10 \| 0.73-6.07 \| 0.168 \| \| T4 \| 2.67 \| 1.10-6.50 \| 0.031 \| \| Lymphovascular Invasion \|  \|  \|  \| \| No \| Reference \| \| \| \| Yes \| 1.65 \| 0.67-4.08 \| 0.279 \| \| LNR (per 0.1 increase) \| 1.33 \| 1.14-1.54 \| <0.001 \| \| CEA \|  \|  \|  \| \| ≤5 ng/mL \| Reference \| \| \| \| >5 ng/mL \| 1.65 \| 0.94-2.89 \| 0.082 \| \| Adjuvant Chemotherapy \|  \|  \|  \| \| No \| Reference \| \| \| \| Yes \| 1.43 \| 0.78-2.60 \| 0.248 \| |
| --- | --- | --- | --- | --- | --- | --- | --- | --- | --- | --- | --- | --- | --- | --- | --- | --- | --- | --- | --- | --- | --- | --- | --- | --- | --- | --- | --- | --- | --- | --- | --- | --- | --- | --- | --- | --- | --- | --- | --- | --- | --- | --- | --- | --- | --- | --- | --- | --- | --- | --- | --- | --- | --- | --- | --- | --- | --- | --- | --- | --- | --- | --- | --- | --- | --- | --- | --- | --- | --- | --- | --- | --- |
| Table S7B. The extended multivariable logistic regression model was fitted in the adequate-follow-up cohort, which included all patients with documented recurrence and recurrence-free patients with follow-up of at least 24 months. This model used the same covariate structure as the primary early-recurrence logistic model, with the additional inclusion of lymphovascular invasion to evaluate the robustness of the primary findings after adjustment for this pathologic variable. Odds ratios (ORs), 95% confidence intervals (CIs), and *P* values are presented. Lymph node ratio was modeled as a continuous variable; for clinical interpretability, its effect is presented per 0.1 absolute increase. The association of lymphovascular invasion should be interpreted as exploratory because this extended model was constructed specifically to address the stability of the primary findings after additional adjustment. |

| **Table S8A. Adequate-follow-up sensitivity analyses using alternative definitions of early recurrence**   \| Variable \| 18 Months \| \| \| 24 Months \| \| \| 30 Months \| \| \| \| --- \| --- \| --- \| --- \| --- \| --- \| --- \| --- \| --- \| --- \| \| OR \| 95% CI \| P *value* \| OR \| 95% CI \| P *value* \| OR \| 95% CI \| P *value* \| \| Tumor Differentiation \|  \|  \|  \|  \|  \|  \|  \|  \|  \| \| Well/Moderate \| Reference \| \| \| Reference \| \| \| Reference \| \| \| \| Poor/Undifferentiated \| 1.52 \| 0.71-3.24 \| 0.279 \| 1.19 \| 0.58-2.44 \| 0.644 \| 1.33 \| 0.68-2.61 \| 0.410 \| \| T Stage \|  \|  \|  \|  \|  \|  \|  \|  \|  \| \| T1-2 \| Reference \| \| \| Reference \| \| \| Reference \| \| \| \| T3 \| 2.19 \| 0.67-7.16 \| 0.193 \| 2.15 \| 0.75-6.20 \| 0.156 \| 1.50 \| 0.56-3.98 \| 0.418 \| \| T4 \| 2.80 \| 0.99-7.90 \| 0.051 \| 2.74 \| 1.13-6.65 \| 0.026 \| 2.14 \| 0.98-4.70 \| 0.057 \| \| LNR (per 0.1 increase) \| 1.34 \| 1.16-1.55 \| <0.001 \| 1.36 \| 1.18-1.57 \| <0.001 \| 1.30 \| 1.13-1.50 \| <0.001 \| \| CEA \|  \|  \|  \|  \|  \|  \|  \|  \|  \| \| ≤5 ng/mL \| Reference \| \| \| Reference \| \| \| Reference \| \| \| \| >5 ng/mL \| 1.55 \| 0.83-2.88 \| 0.166 \| 1.65 \| 0.94-2.88 \| 0.082 \| 1.56 \| 0.91-2.66 \| 0.107 \| \| Adjuvant Chemotherapy \|  \|  \|  \|  \|  \|  \|  \|  \|  \| \| No \| Reference \| \| \| Reference \| \| \| Reference \| \| \| \| Yes \| 1.23 \| 0.63-2.40 \| 0.546 \| 1.39 \| 0.76-2.53 \| 0.281 \| 1.67 \| 0.94-2.95 \| 0.078 \| |
| --- | --- | --- | --- | --- | --- | --- | --- | --- | --- | --- | --- | --- | --- | --- | --- | --- | --- | --- | --- | --- | --- | --- | --- | --- | --- | --- | --- | --- | --- | --- | --- | --- | --- | --- | --- | --- | --- | --- | --- | --- | --- | --- | --- | --- | --- | --- | --- | --- | --- | --- | --- | --- | --- | --- | --- | --- | --- | --- | --- | --- | --- | --- | --- | --- | --- | --- | --- | --- | --- | --- | --- | --- | --- | --- | --- | --- | --- | --- | --- | --- | --- | --- | --- | --- | --- | --- | --- | --- | --- | --- | --- | --- | --- | --- | --- | --- | --- | --- | --- | --- | --- | --- | --- | --- | --- | --- | --- | --- | --- | --- | --- | --- | --- | --- | --- | --- | --- | --- | --- | --- | --- | --- | --- | --- | --- | --- | --- | --- | --- | --- | --- | --- | --- | --- | --- | --- | --- | --- | --- | --- | --- | --- | --- | --- | --- | --- | --- | --- | --- | --- | --- | --- | --- | --- | --- | --- | --- | --- | --- |
| Table S8A. Multivariable logistic regression analyses were repeated using alternative definitions of early recurrence at 18, 24, and 30 months. For each cutoff, early recurrence was defined as documented recurrence occurring within the corresponding postoperative time window. To reduce potential outcome misclassification, recurrence-free patients with follow-up shorter than the corresponding cutoff were excluded, whereas patients with recurrence after the cutoff and recurrence-free patients with follow-up at least as long as the cutoff were classified as non-early recurrence. The same covariate structure as the primary early-recurrence logistic model was used. Odds ratios (ORs), 95% confidence intervals (CIs), and *P* values are presented. Lymph node ratio was modeled as a continuous variable; for clinical interpretability, its effect is presented per 0.1 absolute increase. For consistency, logistic regression confidence intervals and P values were both derived using Wald-based inference. |

| **Table S8B. Full-cohort sensitivity analyses using alternative definitions of early recurrence**   \| Variable \| 18 Months \| \| \| 24 Months \| \| \| 30 Months \| \| \| \| --- \| --- \| --- \| --- \| --- \| --- \| --- \| --- \| --- \| --- \| \| OR \| 95% CI \| P *value* \| OR \| 95% CI \| P *value* \| OR \| 95% CI \| P *value* \| \| Tumor Differentiation \|  \|  \|  \|  \|  \|  \|  \|  \|  \| \| Well/Moderate \| Reference \| \| \| Reference \| \| \| Reference \| \| \| \| Poor/Undifferentiated \| 1.26 \| 0.62-2.56 \| 0.528 \| 1.12 \| 0.59-2.13 \| 0.733 \| 1.33 \| 0.75-2.36 \| 0.335 \| \| T Stage \|  \|  \|  \|  \|  \|  \|  \|  \|  \| \| T1-2 \| Reference \| \| \| Reference \| \| \| Reference \| \| \| \| T3 \| 1.66 \| 0.52-5.26 \| 0.389 \| 1.34 \| 0.48-3.73 \| 0.572 \| 1.04 \| 0.42-2.58 \| 0.939 \| \| T4 \| 3.09 \| 1.13-8.46 \| 0.028 \| 3.29 \| 1.40-7.74 \| 0.006 \| 2.95 \| 1.42-6.14 \| 0.004 \| \| LNR (per 0.1 increase) \| 1.35 \| 1.18-1.54 \| <0.001 \| 1.32 \| 1.17-1.49 \| <0.001 \| 1.26 \| 1.12-1.41 \| <0.001 \| \| CEA \|  \|  \|  \|  \|  \|  \|  \|  \|  \| \| ≤5 ng/mL \| Reference \| \| \| Reference \| \| \| Reference \| \| \| \| >5 ng/mL \| 1.52 \| 0.84-2.77 \| 0.166 \| 1.56 \| 0.93-2.64 \| 0.094 \| 1.52 \| 0.95-2.45 \| 0.084 \| \| Adjuvant Chemotherapy \|  \|  \|  \|  \|  \|  \|  \|  \|  \| \| No \| Reference \| \| \| Reference \| \| \| Reference \| \| \| \| Yes \| 1.30 \| 0.69-2.45 \| 0.417 \| 1.39 \| 0.80-2.43 \| 0.243 \| 1.60 \| 0.97-2.67 \| 0.068 \| |
| --- | --- | --- | --- | --- | --- | --- | --- | --- | --- | --- | --- | --- | --- | --- | --- | --- | --- | --- | --- | --- | --- | --- | --- | --- | --- | --- | --- | --- | --- | --- | --- | --- | --- | --- | --- | --- | --- | --- | --- | --- | --- | --- | --- | --- | --- | --- | --- | --- | --- | --- | --- | --- | --- | --- | --- | --- | --- | --- | --- | --- | --- | --- | --- | --- | --- | --- | --- | --- | --- | --- | --- | --- | --- | --- | --- | --- | --- | --- | --- | --- | --- | --- | --- | --- | --- | --- | --- | --- | --- | --- | --- | --- | --- | --- | --- | --- | --- | --- | --- | --- | --- | --- | --- | --- | --- | --- | --- | --- | --- | --- | --- | --- | --- | --- | --- | --- | --- | --- | --- | --- | --- | --- | --- | --- | --- | --- | --- | --- | --- | --- | --- | --- | --- | --- | --- | --- | --- | --- | --- | --- | --- | --- | --- | --- | --- | --- | --- | --- | --- | --- | --- | --- | --- | --- | --- | --- | --- | --- | --- |
| Table S8B. Multivariable logistic regression analyses were repeated using alternative definitions of early recurrence at 18, 24, and 30 months in the full cohort. In this full-cohort sensitivity analysis, all 1,045 patients were included, and patients without documented recurrence were classified as non-early recurrence regardless of follow-up duration. Because recurrence-free patients with follow-up shorter than the corresponding cutoff may have uncertain early-recurrence status, this analysis was considered supplementary to the adequate-follow-up analyses. Odds ratios (ORs), 95% confidence intervals (CIs), and *P* values are presented. Lymph node ratio was modeled as a continuous variable; for clinical interpretability, its effect is presented per 0.1 absolute increase. For consistency, logistic regression confidence intervals and P values were both derived using Wald-based inference. |

| **Table S9. Internal validation of the primary early-recurrence logistic model and Cox models for TTR and OS**   \| Model \| Early recurrence logistic model \| TTR Cox model \| OS Cox model \| \| --- \| --- \| --- \| --- \| \| Apparent discrimination \| AUC 0.760 \| C-index 0.744 \| C-index 0.779 \| \| Optimism-corrected discrimination \| AUC 0.740 \| C-index 0.731 \| C-index 0.761 \| \| Calibration intercept \| 0.000 \| — \| — \| \| Calibration slope \| 1.000 \| — \| — \| \| Brier score \| 0.101 \| — \| — \| \| Null Brier score \| 0.118 \| — \| — \| \| Scaled Brier score \| 0.144 \| — \| — \| \| Calibration time points \| 24 months \| 24 and 36 months \| 24 and 36 months \| |
| --- | --- | --- | --- | --- | --- | --- | --- | --- | --- | --- | --- | --- | --- | --- | --- | --- | --- | --- | --- | --- | --- | --- | --- | --- | --- | --- | --- | --- | --- | --- | --- | --- | --- | --- | --- | --- |
| Table S9. Internal validation was performed for the primary early-recurrence logistic model and the multivariable Cox models for time-to-recurrence (TTR) and overall survival (OS). The early-recurrence logistic model was validated in the adequate-follow-up cohort, whereas the TTR and OS Cox models were validated in the full cohort. Apparent and optimism-corrected discrimination are reported as the area under the receiver operating characteristic curve (AUC) for the logistic model and the concordance index (C-index) for the Cox models. Calibration intercept, calibration slope, and Brier score are shown for the primary adequate-follow-up early-recurrence logistic model. The null Brier score represents the prediction error of an intercept-only model assigning the observed early-recurrence event probability to all patients in the adequate-follow-up cohort. The scaled Brier score was calculated as 1 minus the ratio of the model Brier score to the null Brier score, with higher values indicating greater relative reduction in prediction error compared with the null model. Time-specific calibration for the Cox models was assessed at 24 and 36 months and is shown in Figure S4. Dashes indicate metrics that were not applicable or not summarized for the corresponding model. Optimism correction was based on 500 bootstrap resamples. |

| **Table S10. Restricted cubic spline analyses of lymph node ratio in relation to early recurrence, time-to-recurrence, and overall survival**   \| Model \| Endpoint \| Cohort \| *N* \| Events \| Knots \| Overall *P* \| *P* for non-linearity \| \| --- \| --- \| --- \| --- \| --- \| --- \| --- \| --- \| \| Multivariable logistic RCS \| Early recurrence \| Adequate-follow-up cohort \| 503 \| 69 \| 0, 0.111, 0.343, 1 \| <0.001 \| 0.971 \| \| Multivariable logistic RCS \| Early recurrence \| Full cohort \| 1045 \| 69 \| 0, 0.111, 0.343, 1 \| <0.001 \| 0.935 \| \| Multivariable Cox RCS \| TTR \| Full cohort \| 1045 \| 106 \| 0, 0.111, 0.343, 1 \| <0.001 \| 0.706 \| \| Multivariable Cox RCS \| OS \| Full cohort \| 1045 \| 86 \| 0, 0.111, 0.343, 1 \| <0.001 \| 0.441 \| |
| --- | --- | --- | --- | --- | --- | --- | --- | --- | --- | --- | --- | --- | --- | --- | --- | --- | --- | --- | --- | --- | --- | --- | --- | --- | --- | --- | --- | --- | --- | --- | --- | --- | --- | --- | --- | --- | --- | --- | --- | --- |
| Table S10. Restricted cubic spline analyses were performed to evaluate the overall and nonlinear associations of lymph node ratio (LNR) with early recurrence, time-to-recurrence (TTR), and overall survival (OS). For early recurrence, the primary spline model was fitted in the adequate-follow-up cohort, which included all patients with documented recurrence and recurrence-free patients with follow-up of at least 24 months. The full-cohort early-recurrence spline model was retained as a sensitivity analysis. For TTR and OS, spline analyses were performed in the full cohort using multivariable Cox models. Models were adjusted using the same covariate structure as the corresponding primary regression models. LNR was retained on its original 0-1 scale for spline modeling and visualization; regression estimates in the main and supplementary tables are presented per 0.1 absolute increase for clinical interpretability. Because LNR was zero-inflated in this cohort, with 682 of 1,045 patients having LNR = 0, knots were selected using a prespecified robust knot-selection algorithm. The algorithm first attempted empirical quantile-based knots, including the 5th, 35th, 65th, and 95th percentiles and predefined alternative quantile sets. Because these quantile-based rules produced duplicate knots due to the high frequency of LNR = 0, the fallback rule selected four non-duplicated knot values from approximately equally spaced positions of the sorted unique LNR values. This procedure yielded knots at 0, 0.111, 0.343, and 1, which were used consistently across all endpoints and cohorts for comparability. The overall P value tests whether LNR is associated with the endpoint overall, whereas the P for nonlinearity tests whether the association significantly deviates from linearity. The spline analyses were exploratory assessments of functional form and were not intended to derive a clinical threshold. |

| **Table S11. Distribution patterns and event-time characteristics of lymphovascular invasion in the full cohort**   \| Variable \| Category \| LVI No (n=639) \| LVI Yes (n=406) \| \| --- \| --- \| --- \| --- \| \| Part A. Composition differences \| \| \| \| \| TTR event \| No \| 552 (86.4%) \| 387 (95.3%) \| \|  \| Yes \| 87 (13.6%) \| 19 (4.7%) \| \| Early recurrence (≤24 months) \| No early recurrence \| 583 (91.2%) \| 393 (96.8%) \| \|  \| Early recurrence \| 56 (8.8%) \| 13 (3.2%) \| \| T stage \| T1-2 \| 230 (36.0%) \| 116 (28.6%) \| \|  \| T3 \| 118 (18.5%) \| 126 (31.0%) \| \|  \| T4 \| 291 (45.5%) \| 164 (40.4%) \| \| N stage \| N0 \| 414 (64.8%) \| 235 (57.9%) \| \|  \| N1 \| 162 (25.4%) \| 111 (27.3%) \| \|  \| N2 \| 63 (9.9%) \| 60 (14.8%) \| \| AJCC stage \| I \| 200 (31.3%) \| 96 (23.6%) \| \|  \| II \| 214 (33.5%) \| 139 (34.2%) \| \|  \| III \| 225 (35.2%) \| 171 (42.1%) \| \| Adjuvant chemotherapy \| No \| 384 (60.1%) \| 237 (58.4%) \| \|  \| Yes \| 255 (39.9%) \| 169 (41.6%) \| \| Part B. Event-time distribution among patients with events \| \| \| \| \| TTR Months among patients with TTR_Event = 1 \|  \| 19.0 [12.5-28.5] \| 13.0 [6.0-30.0] \| \| OS Months among patients with OS_Event = 1 \|  \| 26.0 [15.0-40.0] \| 20.0 [9.0-35.0] \| |
| --- | --- | --- | --- | --- | --- | --- | --- | --- | --- | --- | --- | --- | --- | --- | --- | --- | --- | --- | --- | --- | --- | --- | --- | --- | --- | --- | --- | --- | --- | --- | --- | --- | --- | --- | --- | --- | --- | --- | --- | --- | --- | --- | --- | --- | --- | --- | --- | --- | --- | --- | --- | --- | --- | --- | --- | --- | --- | --- | --- | --- | --- | --- | --- | --- | --- | --- | --- | --- | --- | --- | --- | --- | --- | --- | --- | --- | --- | --- | --- | --- |
| Table S11. Values in Part A are presented as n (%), and values in Part B as median [interquartile range]. Early recurrence was defined as recurrence within 24 months after surgery. *P* values in Part A were calculated using Pearson’s chi-square test or Fisher’s exact test, as appropriate, and *P* values in Part B were calculated using the Wilcoxon rank-sum test. This table was constructed to examine whether the apparent instability of lymphovascular invasion across different regression models could be explained by coding error, compositional differences in stage or treatment, or heterogeneity in event timing. |

| **Table S12. Pairwise Spearman rank correlations among candidate predictors considered for multivariable modeling**   \| Variable \| T Stage \| N Stage \| AJCC Stage \| LNR \| Tumor Size (cm) \| Tumor Differentiation \| LVI \| CEA \| CA19-9 \| Adjuvant Chemotherapy \| Neoadjuvant Therapy \| \| --- \| --- \| --- \| --- \| --- \| --- \| --- \| --- \| --- \| --- \| --- \| --- \| \| T Stage \| 1.00 \| 0.39 \| 0.64 \| 0.37 \| 0.37 \| 0.18 \| 0.01 \| 0.21 \| 0.19 \| 0.31 \| -0.03 \| \| N Stage \| 0.39 \| 1.00 \| 0.87 \| 0.93 \| 0.18 \| 0.21 \| 0.08 \| 0.17 \| 0.18 \| 0.54 \| 0.01 \| \| AJCC Stage \| 0.64 \| 0.87 \| 1.00 \| 0.81 \| 0.31 \| 0.19 \| 0.09 \| 0.20 \| 0.17 \| 0.55 \| 0.01 \| \| LNR \| 0.37 \| 0.93 \| 0.81 \| 1.00 \| 0.19 \| 0.21 \| 0.06 \| 0.18 \| 0.19 \| 0.51 \| -0.03 \| \| Tumor Size (cm) \| 0.37 \| 0.18 \| 0.31 \| 0.19 \| 1.00 \| 0.10 \| 0.03 \| 0.22 \| 0.11 \| 0.16 \| -0.05 \| \| Tumor Differentiation \| 0.18 \| 0.21 \| 0.19 \| 0.21 \| 0.10 \| 1.00 \| 0.10 \| 0.03 \| 0.14 \| 0.14 \| 0.09 \| \| LVI \| 0.01 \| 0.08 \| 0.09 \| 0.06 \| 0.03 \| 0.10 \| 1.00 \| -0.05 \| 0.02 \| 0.02 \| 0.07 \| \| CEA \| 0.21 \| 0.17 \| 0.20 \| 0.18 \| 0.22 \| 0.03 \| -0.05 \| 1.00 \| 0.20 \| 0.10 \| -0.05 \| \| CA19-9 \| 0.19 \| 0.18 \| 0.17 \| 0.19 \| 0.11 \| 0.14 \| 0.02 \| 0.20 \| 1.00 \| 0.04 \| -0.03 \| \| Adjuvant Chemotherapy \| 0.31 \| 0.54 \| 0.55 \| 0.51 \| 0.16 \| 0.14 \| 0.02 \| 0.10 \| 0.04 \| 1.00 \| 0.16 \| \| Neoadjuvant Therapy \| -0.03 \| 0.01 \| 0.01 \| -0.03 \| -0.05 \| 0.09 \| 0.07 \| -0.05 \| -0.03 \| 0.16 \| 1.00 \| |
| --- | --- | --- | --- | --- | --- | --- | --- | --- | --- | --- | --- | --- | --- | --- | --- | --- | --- | --- | --- | --- | --- | --- | --- | --- | --- | --- | --- | --- | --- | --- | --- | --- | --- | --- | --- | --- | --- | --- | --- | --- | --- | --- | --- | --- | --- | --- | --- | --- | --- | --- | --- | --- | --- | --- | --- | --- | --- | --- | --- | --- | --- | --- | --- | --- | --- | --- | --- | --- | --- | --- | --- | --- | --- | --- | --- | --- | --- | --- | --- | --- | --- | --- | --- | --- | --- | --- | --- | --- | --- | --- | --- | --- | --- | --- | --- | --- | --- | --- | --- | --- | --- | --- | --- | --- | --- | --- | --- | --- | --- | --- | --- | --- | --- | --- | --- | --- | --- | --- | --- | --- | --- | --- | --- | --- | --- | --- | --- | --- | --- | --- | --- | --- | --- | --- | --- | --- | --- | --- | --- | --- | --- | --- | --- | --- |
| Table S12. Pairwise Spearman rank correlation coefficients are shown for candidate predictors considered during multivariable model construction. Ordinal variables were numerically encoded according to their natural order, and binary variables were encoded as 0/1 for correlation assessment. Correlations were used descriptively to evaluate potential collinearity rather than as formal criteria for variable inclusion. Strong correlations were observed among N stage, AJCC stage, and LNR, supporting the decision not to include N stage or AJCC stage simultaneously with LNR in the primary multivariable models. Adjuvant chemotherapy showed moderate correlation with nodal and stage-related variables and was therefore interpreted as an adjustment covariate rather than as a causal treatment effect. |

| **Table S13. Endpoint-specific model construction and covariate-selection rationale**   \| Endpoint / model \| Analysis cohort \| Candidate predictors \| Selection principle \| Final covariates \| Variables not retained in the final model \| Rationale \| \| --- \| --- \| --- \| --- \| --- \| --- \| --- \| \| Early-recurrence logistic model \| Adequate-follow-up cohort \| Baseline clinicopathological and treatment variables listed in Methods \| Univariable P < 0.10, clinical relevance, Spearman correlation assessment, and model parsimony \| LNR, T stage, CEA, tumor differentiation, adjuvant chemotherapy \| N stage, AJCC stage \| N stage and AJCC stage were not modeled together with LNR because of strong correlations among nodal/stage variables. LNR was retained as a more granular continuous indicator of nodal tumor burden. Adjuvant chemotherapy was included as an adjustment covariate rather than interpreted causally. \| \| Extended early-recurrence logistic model including LVI \| Adequate-follow-up cohort \| Same as primary early-recurrence logistic model \| Supplementary robustness analysis \| LNR, T stage, CEA, tumor differentiation, adjuvant chemotherapy, LVI \| N stage, AJCC stage \| This model was constructed to evaluate whether the primary findings were robust to additional adjustment for LVI. \| \| TTR Cox model \| Full cohort \| Baseline clinicopathological and treatment variables listed in Methods \| Univariable P < 0.10, clinical relevance, Spearman correlation assessment, event-number considerations, and consistency with the OS model \| Tumor size, tumor differentiation, T stage, LVI, LNR, CEA, adjuvant chemotherapy \| N stage, AJCC stage \| The model evaluated recurrence-focused time-to-event risk. LNR was retained instead of N stage/AJCC stage to reduce collinearity while preserving continuous nodal-burden information. The TTR and OS Cox models used the same covariate set for consistency across long-term time-to-event endpoints. \| \| OS Cox model \| Full cohort \| Baseline clinicopathological and treatment variables listed in Methods \| Same as TTR Cox model \| Tumor size, tumor differentiation, T stage, LVI, LNR, CEA, adjuvant chemotherapy \| N stage, AJCC stage \| The OS Cox model used the same covariate set as the TTR Cox model to improve transparency and comparability across long-term outcome analyses. \| \| Fine–Gray model for distant metastasis \| Full cohort \| Baseline clinicopathological and treatment variables listed in Methods \| Clinical relevance, collinearity assessment, endpoint specificity, and event-number considerations \| Tumor differentiation, T stage, LNR, CEA, adjuvant chemotherapy \| N stage, AJCC stage, LVI, tumor size \| The model focused on distant metastasis under a competing-risk framework. A parsimonious core covariate set was used to avoid overfitting and to maintain consistency with the main prognostic framework. \| \| Fine–Gray model for local recurrence \| Full cohort \| Baseline clinicopathological and treatment variables listed in Methods \| Exploratory analysis because of limited local-recurrence events \| Tumor differentiation, T stage, LNR, CEA, adjuvant chemotherapy \| N stage, AJCC stage, LVI, tumor size \| Because only 24 local recurrence events occurred, the multivariable local-recurrence model was interpreted as exploratory. The same core covariate set as the distant-metastasis model was used for comparability. \| |
| --- | --- | --- | --- | --- | --- | --- | --- | --- | --- | --- | --- | --- | --- | --- | --- | --- | --- | --- | --- | --- | --- | --- | --- | --- | --- | --- | --- | --- | --- | --- | --- | --- | --- | --- | --- | --- | --- | --- | --- | --- | --- | --- | --- | --- | --- | --- | --- | --- | --- |
| Table S13. This table summarizes endpoint-specific model construction and covariate-selection rationale. Candidate predictors included the baseline clinicopathological and treatment variables listed in the Methods. Final covariates were selected according to univariable screening, clinical relevance, Spearman correlation assessment, collinearity considerations, model parsimony, and event-number constraints. AJCC stage and N stage were not modeled simultaneously with LNR because of strong correlations among nodal and stage-related variables. Adjuvant chemotherapy was included as an adjustment covariate to account for treatment allocation associated with baseline risk and was not interpreted as a causal treatment effect. |

| **Table S14A. Baseline characteristics of the adequate-follow-up cohort and excluded short-follow-up recurrence-free patients**   \| Variable \| Adequate-follow-up cohort (n=503) \| Excluded recurrence-free patients with follow-up <24 months (n = 542) \| P value \| \| --- \| --- \| --- \| --- \| \| Age of Diagnosis \| 60.00 [51.00, 68.00] \| 60.00 [52.25, 67.00] \| 0.871 \| \| BMI \| 22.00 [20.00, 24.00] \| 22.00 [20.00, 24.00] \| 0.274 \| \| Tumor Size (cm) \| 3.00 [3.00, 4.00] \| 3.00 [3.00, 4.00] \| 0.695 \| \| Distance to Dentate Line (cm) \| 6.00 [3.00, 10.00] \| 5.00 [2.00, 8.00] \| <0.001 \| \| Lymph Node Ratio \| 0.00 [0.00, 0.10] \| 0.00 [0.00, 0.07] \| 0.096 \| \| Sex \|  \|  \| 1.000 \| \| Female \| 194 (38.6) \| 208 (38.4) \|  \| \| Male \| 309 (61.4) \| 334 (61.6) \|  \| \| Histology Type \|  \|  \| 0.799 \| \| Adenocarcinoma \| 459 (91.3) \| 498 (91.9) \|  \| \| Mucinous/Signet Ring \| 44 (8.7) \| 44 (8.1) \|  \| \| Tumor Differentiation \|  \|  \| 0.899 \| \| Well/Moderate \| 432 (85.9) \| 468 (86.3) \|  \| \| Poor/Undifferentiated \| 71 (14.1) \| 74 (13.7) \|  \| \| Lymphovascular Invasion \|  \|  \| <0.001 \| \| No \| 469 (93.2) \| 170 (31.4) \|  \| \| Yes \| 34 (6.8) \| 372 (68.6) \|  \| \| Surgical Approach \|  \|  \| 0.003 \| \| Open \| 175 (34.8) \| 141 (26.0) \|  \| \| Laparoscopic \| 328 (65.2) \| 401 (74.0) \|  \| \| Sphincter Preservation \|  \|  \| 0.272 \| \| No \| 125 (24.9) \| 152 (28.0) \|  \| \| Yes \| 378 (75.1) \| 390 (72.0) \|  \| \| TME Performed \|  \|  \| 0.104 \| \| No \| 166 (33.0) \| 206 (38.0) \|  \| \| Yes \| 337 (67.0) \| 336 (62.0) \|  \| \| CEA \|  \|  \| 0.776 \| \| ≤5 ng/mL \| 328 (65.2) \| 359 (66.2) \|  \| \| >5 ng/mL \| 175 (34.8) \| 183 (33.8) \|  \| \| CA19-9 \|  \|  \| 0.472 \| \| ≤35 U/mL \| 445 (88.5) \| 488 (90.0) \|  \| \| >35 U/mL \| 58 (11.5) \| 54 (10.0) \|  \| \| Neoadjuvant Therapy \|  \|  \| 0.016 \| \| No \| 466 (92.6) \| 477 (88.0) \|  \| \| Yes \| 37 (7.4) \| 65 (12.0) \|  \| \| Adjuvant Chemotherapy \|  \|  \| 0.495 \| \| No \| 293 (58.3) \| 328 (60.5) \|  \| \| Yes \| 210 (41.7) \| 214 (39.5) \|  \| \| T Stage \|  \|  \| <0.001 \| \| T1-2 \| 166 (33.0) \| 180 (33.2) \|  \| \| T3 \| 79 (15.7) \| 165 (30.4) \|  \| \| T4 \| 258 (51.3) \| 197 (36.3) \|  \| \| N Stage \|  \|  \| 0.402 \| \| N0 \| 310 (61.6) \| 339 (62.5) \|  \| \| N1 \| 127 (25.2) \| 146 (26.9) \|  \| \| N2 \| 66 (13.1) \| 57 (10.5) \|  \| \| AJCC Stage \|  \|  \| 0.950 \| \| I \| 142 (28.2) \| 154 (28.4) \|  \| \| II \| 168 (33.4) \| 185 (34.1) \|  \| \| III \| 193 (38.4) \| 203 (37.5) \|  \| |
| --- | --- | --- | --- | --- | --- | --- | --- | --- | --- | --- | --- | --- | --- | --- | --- | --- | --- | --- | --- | --- | --- | --- | --- | --- | --- | --- | --- | --- | --- | --- | --- | --- | --- | --- | --- | --- | --- | --- | --- | --- | --- | --- | --- | --- | --- | --- | --- | --- | --- | --- | --- | --- | --- | --- | --- | --- | --- | --- | --- | --- | --- | --- | --- | --- | --- | --- | --- | --- | --- | --- | --- | --- | --- | --- | --- | --- | --- | --- | --- | --- | --- | --- | --- | --- | --- | --- | --- | --- | --- | --- | --- | --- | --- | --- | --- | --- | --- | --- | --- | --- | --- | --- | --- | --- | --- | --- | --- | --- | --- | --- | --- | --- | --- | --- | --- | --- | --- | --- | --- | --- | --- | --- | --- | --- | --- | --- | --- | --- | --- | --- | --- | --- | --- | --- | --- | --- | --- | --- | --- | --- | --- | --- | --- | --- | --- | --- | --- | --- | --- | --- | --- | --- | --- | --- | --- | --- | --- | --- | --- | --- | --- | --- | --- | --- | --- | --- | --- | --- | --- | --- | --- | --- | --- | --- | --- | --- | --- | --- | --- | --- | --- | --- | --- | --- | --- | --- | --- | --- | --- | --- | --- | --- | --- | --- | --- | --- | --- | --- | --- | --- | --- | --- | --- | --- |
| Table S14A. Baseline characteristics were compared between the adequate-follow-up cohort used for the primary early-recurrence logistic analysis and recurrence-free patients excluded because of follow-up shorter than 24 months. The adequate-follow-up cohort included all patients with documented recurrence and recurrence-free patients with follow-up of at least 24 months. Therefore, this comparison was intended to characterize the overall analytic restriction rather than to compare two recurrence-free groups. Continuous variables are presented as median [interquartile range], and categorical variables are presented as number (column percentage). P values are descriptive and were calculated using the Wilcoxon rank-sum test for continuous variables and Pearson’s chi-square test or Fisher’s exact test for categorical variables, as appropriate. Follow-up duration was not compared because it defined exclusion status. |

| **Table S14B. Baseline characteristics of retained and excluded recurrence-free patients according to follow-up adequacy**   \| Variable \| Retained recurrence-free patients with follow-up ≥24 months (n = 397) \| Excluded recurrence-free patients with follow-up <24 months (n = 542) \| P value \| \| --- \| --- \| --- \| --- \| \| Age of Diagnosis \| 60.00 [51.00, 68.00] \| 60.00 [52.25, 67.00] \| 0.898 \| \| BMI \| 22.00 [20.00, 24.00] \| 22.00 [20.00, 24.00] \| 0.085 \| \| Tumor Size (cm) \| 3.00 [3.00, 4.00] \| 3.00 [3.00, 4.00] \| 0.175 \| \| Distance to Dentate Line (cm) \| 6.00 [3.00, 10.00] \| 5.00 [2.00, 8.00] \| <0.001 \| \| Lymph Node Ratio \| 0.00 [0.00, 0.06] \| 0.00 [0.00, 0.07] \| 0.286 \| \| Sex \|  \|  \|  \| \| Female \| 155 (39.0) \| 208 (38.4) \| 0.889 \| \| Male \| 242 (61.0) \| 334 (61.6) \|  \| \| Histology Type \|  \|  \|  \| \| Adenocarcinoma \| 366 (92.2) \| 498 (91.9) \| 0.959 \| \| Mucinous/Signet Ring \| 31 (7.8) \| 44 (8.1) \|  \| \| Tumor Differentiation \|  \|  \|  \| \| Well/Moderate \| 353 (88.9) \| 468 (86.3) \| 0.283 \| \| Poor/Undifferentiated \| 44 (11.1) \| 74 (13.7) \|  \| \| Lymphovascular Invasion \|  \|  \|  \| \| No \| 382 (96.2) \| 170 (31.4) \| <0.001 \| \| Yes \| 15 (3.8) \| 372 (68.6) \|  \| \| Surgical Approach \|  \|  \|  \| \| Open \| 140 (35.3) \| 141 (26.0) \| 0.003 \| \| Laparoscopic \| 257 (64.7) \| 401 (74.0) \|  \| \| Sphincter Preservation \|  \|  \|  \| \| No \| 93 (23.4) \| 152 (28.0) \| 0.129 \| \| Yes \| 304 (76.6) \| 390 (72.0) \|  \| \| TME Performed \|  \|  \|  \| \| No \| 125 (31.5) \| 206 (38.0) \| 0.046 \| \| Yes \| 272 (68.5) \| 336 (62.0) \|  \| \| CEA \|  \|  \|  \| \| ≤5 ng/mL \| 278 (70.0) \| 359 (66.2) \| 0.247 \| \| >5 ng/mL \| 119 (30.0) \| 183 (33.8) \|  \| \| CA19-9 \|  \|  \|  \| \| ≤35 U/mL \| 359 (90.4) \| 488 (90.0) \| 0.930 \| \| >35 U/mL \| 38 (9.6) \| 54 (10.0) \|  \| \| Neoadjuvant Therapy \|  \|  \|  \| \| No \| 369 (92.9) \| 477 (88.0) \| 0.017 \| \| Yes \| 28 (7.1) \| 65 (12.0) \|  \| \| Adjuvant Chemotherapy \|  \|  \|  \| \| No \| 255 (64.2) \| 328 (60.5) \| 0.275 \| \| Yes \| 142 (35.8) \| 214 (39.5) \|  \| \| T Stage \|  \|  \|  \| \| T1-2 \| 154 (38.8) \| 180 (33.2) \| <0.001 \| \| T3 \| 67 (16.9) \| 165 (30.4) \|  \| \| T4 \| 176 (44.3) \| 197 (36.3) \|  \| \| N Stage \|  \|  \|  \| \| N0 \| 276 (69.5) \| 339 (62.5) \| 0.070 \| \| N1 \| 83 (20.9) \| 146 (26.9) \|  \| \| N2 \| 38 (9.6) \| 57 (10.5) \|  \| \| AJCC Stage \|  \|  \|  \| \| I \| 132 (33.2) \| 154 (28.4) \| 0.071 \| \| II \| 144 (36.3) \| 185 (34.1) \|  \| \| III \| 121 (30.5) \| 203 (37.5) \|  \| |
| --- | --- | --- | --- | --- | --- | --- | --- | --- | --- | --- | --- | --- | --- | --- | --- | --- | --- | --- | --- | --- | --- | --- | --- | --- | --- | --- | --- | --- | --- | --- | --- | --- | --- | --- | --- | --- | --- | --- | --- | --- | --- | --- | --- | --- | --- | --- | --- | --- | --- | --- | --- | --- | --- | --- | --- | --- | --- | --- | --- | --- | --- | --- | --- | --- | --- | --- | --- | --- | --- | --- | --- | --- | --- | --- | --- | --- | --- | --- | --- | --- | --- | --- | --- | --- | --- | --- | --- | --- | --- | --- | --- | --- | --- | --- | --- | --- | --- | --- | --- | --- | --- | --- | --- | --- | --- | --- | --- | --- | --- | --- | --- | --- | --- | --- | --- | --- | --- | --- | --- | --- | --- | --- | --- | --- | --- | --- | --- | --- | --- | --- | --- | --- | --- | --- | --- | --- | --- | --- | --- | --- | --- | --- | --- | --- | --- | --- | --- | --- | --- | --- | --- | --- | --- | --- | --- | --- | --- | --- | --- | --- | --- | --- | --- | --- | --- | --- | --- | --- | --- | --- | --- | --- | --- | --- | --- | --- | --- | --- | --- | --- | --- | --- | --- | --- | --- | --- | --- | --- | --- | --- | --- | --- | --- | --- | --- | --- | --- | --- | --- | --- | --- | --- | --- | --- |
| Table S14B. To more directly assess potential selection among recurrence-free patients, baseline characteristics were compared between recurrence-free patients retained in the adequate-follow-up cohort and recurrence-free patients excluded because of follow-up shorter than 24 months. Retained recurrence-free patients were those without documented recurrence and with follow-up of at least 24 months; excluded recurrence-free patients were those without documented recurrence and with follow-up shorter than 24 months. Continuous variables are presented as median [interquartile range], and categorical variables are presented as number (column percentage). P values are descriptive and were calculated using the Wilcoxon rank-sum test for continuous variables and Pearson’s chi-square test or Fisher’s exact test for categorical variables, as appropriate. Follow-up duration was not compared because it defined the grouping. |

**Supplementary Statistical Appendix. Hazard smoothing and model specification notes**

**1. Hazard-smoothing specification**

The smoothed postoperative recurrence hazard was estimated in the full cohort using TTR months as the time scale and TTR event as the recurrence indicator. Patients without documented recurrence were treated as right-censored at death or last follow-up. Hazard smoothing was performed using a nonparametric B-spline approach implemented in R version 4.5.2 with the bshazard package, using the bshazard function. The smoothing specification was nbin = 60, degree = 3, lambda = NULL (default setting). The primary hazard peak was identified as the dominant local maximum of the smoothed recurrence-hazard curve within the clinically interpretable follow-up window. Because the number of patients remaining at risk decreased during late follow-up, the late tail of the hazard curve was interpreted cautiously. The 24-month cutoff was therefore considered an exploratory, data-informed threshold rather than a definitive universal cutoff.

**2. LNR scaling**

LNR was entered into regression models as a continuous variable on its original 0–1 scale. For clinical interpretability, regression estimates were rescaled to represent each 0.1 absolute increase in LNR using the transformation exp[0.1 × log(estimate)]. The same transformation was applied to confidence interval limits, and P values were unchanged. LNR was retained on the original scale for graphical stratification and spline visualization.

**3. Logistic regression inference**

For logistic regression models, confidence intervals and P values were both derived using Wald-based inference to ensure consistency between interval estimates and significance testing. This approach was applied consistently to the primary early-recurrence logistic model and the supplementary logistic sensitivity analyses.

**4. Predictor collinearity assessment**

Pairwise Spearman rank correlations among candidate predictors were calculated descriptively to assess potential collinearity. Strong correlations were observed among N stage, AJCC stage, and LNR, supporting the decision not to include N stage or AJCC stage simultaneously with LNR in the primary multivariable models. Adjuvant chemotherapy showed moderate correlation with nodal and stage-related variables, supporting its interpretation as an adjustment covariate reflecting baseline risk and treatment allocation rather than as a causal treatment effect.

**5. Endpoint-specific model construction**

Multivariable models were constructed according to endpoint-specific inferential aims. The early-recurrence logistic model focused on binary recurrence within the postoperative high-risk window and was fitted in the adequate-follow-up cohort. The TTR and OS Cox models evaluated long-term time-to-event outcomes in the full cohort and used a shared covariate structure to improve comparability. Fine–Gray models evaluated recurrence patterns under a competing-risk framework and used a parsimonious core covariate set because of event-number considerations, particularly for local recurrence.

**6. Restricted cubic spline knot selection**

RCS analyses were used to explore the functional form of the association between LNR and study endpoints, rather than to derive clinical thresholds. Because LNR was zero-inflated in this cohort, with 682 of 1,045 patients having LNR = 0, a prespecified robust knot-selection algorithm was used. The algorithm first attempted empirical quantile-based knots, including the 5th, 35th, 65th, and 95th percentiles and predefined alternative quantile sets. When these quantile-based rules produced duplicate knots, the fallback rule selected four non-duplicated knot values from approximately equally spaced positions of the sorted unique LNR values. This yielded knots at 0, 0.111, 0.343, and 1, which were used consistently across all endpoints and cohorts for comparability.

**7. Decision-curve analysis**

Exploratory decision-curve analyses were performed for the primary adequate-follow-up early-recurrence logistic model and the full-cohort sensitivity model. Net benefit was evaluated across threshold probabilities and compared with treat-all and treat-none strategies, as well as single-variable models based on LNR, T stage, and CEA. These analyses were intended to assess potential clinical utility and were not considered sufficient to establish clinical implementation without external validation.
